# Running exercise mitigates amyloidosis in 5xFAD mice by improving the structure and function of the meningeal lymphatic system

**DOI:** 10.1186/s40478-025-02218-2

**Published:** 2026-01-15

**Authors:** Onanong Mee-inta, Yu-Yi Chiang, Sheng-Feng Tsai, Tzu-Mo Yang, Zi-Wei Zhao, Tzu-Feng Wang, Hsin-Yi Wu, Chien-Wei Hsiung, Pao-Chi Liao, Hsueh-Te Lee, Chih-Chung Huang, Ping-Ching Wu, Yu-Min Kuo

**Affiliations:** 1 Chakri Naruebodindra Medical Institute, Faculty of Medicine Ramathibodi Hospital, Mahidol University, Samut Prakan, Thailand; 2 Institute of Basic Medical Sciences, College of Medicine, National Cheng Kung University, Tainan, Taiwan; 3 Department of Biomedical Engineering, College of Engineering, National Cheng Kung University, Tainan, Taiwan; 4 Department of Cell Biology and Anatomy, College of Medicine, National Cheng Kung University, Tainan, Taiwan; 5 Instrumentation Center, National Taiwan University, Taipei, Taiwan; 6 Department of Environmental and Occupational Health, College of Medicine, National Cheng Kung University, Tainan, Taiwan; 7 Institute of Anatomy and Cell Biology, School of Medicine, National Yang Ming Chiao Tung University, Taipei, Taiwan; 8 Medical Device Innovation Center, National Cheng Kung University, Tainan, Taiwan; 9 Institute of Oral Medicine and Department of Stomatology, National Cheng Kung University Hospital, College of Medicine, National Cheng Kung University, Tainan, Taiwan; 10 Center of Applied Nanomedicine, National Cheng Kung University, Tainan, Taiwan

**Keywords:** Alzheimer’s disease, Amyloidosis, Meningeal lymphatic system, Lymphangiogenesis, Running exercise

## Abstract

**Background:**

Alzheimer’s disease (AD) progression is closely linked to the accumulation of amyloid-$\upbeta$ (A$\upbeta$), with impaired clearance mechanisms playing a key role. The meningeal lymphatic (mLym) system, which drains cerebrospinal fluid (CSF) and waste from the brain to peripheral lymph nodes, has emerged as a critical pathway for A$\upbeta$ removal. While physical exercise is known to improve cognitive function and reduce AD risk, its effect on the mLym system and downstream AD pathology have not been fully elucidated.

**Methods:**

Three-month-old 5xFAD mice underwent a 3-month wheel-running exercise regimen. The function of the mLym system was assessed before and after exercise using high-frequency ultrasound imaging with nanoparticle tracers to monitor CSF drainage to deep cervical lymph nodes. The study evaluated changes in mLym vessel structure, A$\upbeta$ deposition, and cognitive performance. Additionally, the effects of serum and extracellular vesicles (EVs) from exercised rats on the expression of lymphatic vessel-related genes (LYVE-1, VEGFR3, and VEGF-C) were examined in lymphatic endothelial and microglial cell lines.

**Results:**

Compared to 3-month-old 5xFAD mice and age-matched wild-type controls, 6-month-old 5xFAD mice displayed progressive decline in mLym function, reduced vessel integrity, and increased amyloid plaque burden, accompanied by impaired learning and memory. These changes were associated with decreased expression of LYVE-1 and VEGFR3 in the meninges and VEGF-C in the brain. Exercise intervention reversed these deficits, restoring mLym function and vessel structure, enhancing A$\upbeta$ clearance, and improving cognitive performance. Surgical ligation of mLym vessels accelerated amyloid accumulation and removed the exercise-induced benefits, underscoring the system’s importance in A$\upbeta$ removal. *In vitro*, A$\upbeta$ oligomers suppressed VEGFR3 and VEGF-C expression, while serum and EVs from exercised rats counteracted this effect. Proteomic analysis of EVs from exercised animals revealed upregulation of CD9, suggesting a link to VEGFR3 signaling.

**Conclusions:**

This study demonstrates that A$\upbeta$ oligomers impair mLym function, exacerbating amyloid pathology. Exercise preserves the structure and function of the mLym system, promoting A$\upbeta$ clearance and mitigating AD progression. These findings highlight the therapeutic potential of targeting the meningeal lymphatic system to slow or prevent AD.

**Supplementary Information:**

The online version contains supplementary material available at 10.1186/s40478-025-02218-2.

## Background

Alzheimer’s disease (AD) is the most common form of dementia in the elderly. It is widely thought that the pathological progression of AD involves extensive buildup of amyloid plaques, which evolve from amyloid-$\upbeta$ (A$\upbeta$) monomers that progressively aggregate as oligomers and protofibrils before forming mature fibers [1, 2]. A$\upbeta$ may also accumulate along the parenchymal and meningeal blood vessels, resulting in cerebral amyloid angiopathy [3]. According to the ‘ATN’ (amyloid, tau, neurodegeneration) research framework put forward by the National Institute on Aging and the Alzheimer’s Association, the accumulation of A$\upbeta$ triggers a sequence of pathological events that disrupt the neural network and impair synaptic function, finally resulting in the development of AD [4, 5].

Soluble A$\upbeta$ pools are influenced by synthesis, clearance, and aggregation dynamics. Metabolic labeling revealed comparable $\text {A}\upbeta _{\text {1-40}}$ and $\text {A}\upbeta _{\text {1-42}}$ production rates but reduced clearance in late-onset AD patients [6], indicating impaired A$\upbeta$ clearance contributes to A$\upbeta$ accumulation in AD. Early research suggested that the blood-brain barrier is the primary route of A$\upbeta$ clearance [7]. However, more recent studies suggest that the glymphatic system may also be a crucial elimination pathway, transporting A$\upbeta$ from the brain interstitial fluid into the cerebrospinal fluid (CSF) [4, 5, 7]. Subsequently, the CSF is conveyed to the extracranial lymph nodes through the meningeal lymphatic (mLym) vasculature [4, 5, 7]. The mLym vessels are in the dura mater of the meningeal compartment and are known to facilitate transport of fluid, immune cells, and waste products between the brain and the cervical lymph nodes.

Anatomically, mLym vessels comprise dorsal and basal routes [4, 5, 7]. The dorsal route is located near the dural blood vessels along the cribriform plate, superior sagittal sinus (SSS), and transverse sinus (TS). Meanwhile, the basal route is located at the base of the skull, near various bony structures, blood vessels and cranial nerves, exiting the skull to drain mainly into the deep cervical lymph nodes (dCLNs). It has been demonstrated that the capacity for waste clearance is decreased in aged humans and rodents, including the clearance of A$\upbeta$ from the brain to the dCLNs [8, 9]. In addition, glymphatic influx and A$\upbeta$ clearance are both decreased in AD mouse models, while ablation of mLym drainage in AD transgenic mice results in more severe amyloid pathology in the brain and meninges [8, 10]. Moreover, intracisternal injection of $\text {A}\upbeta _{\text {1-40}}$ in wild-type mice inhibits the flow of CSF, indicating that A$\upbeta$ can disrupt the glymphatic system [10].

Clinical studies have shown that AD pathogenesis can be delayed through both pharmacological and non-pharmacological interventions, such as monoclonal antibody therapies and non-invasive brain stimulation [11]. Physical exercise is also recognized as a valuable intervention for preventing or delaying AD. For instance, one long-term study revealed a negative correlation between high levels of physical activity and the onset of cognitive impairment in AD [12]. It was also shown that physical activity can reduce approximately one-third of the risk factors associated with AD, indicating its potential to protect against the disease [13]. In AD mouse models with human mutant APP overexpression, running exercise decreases the accumulation of A$\upbeta$ in the amygdala and hippocampus without altering the levels of APP, and it also mitigates associated neurodegeneration [14]. Moreover, AD mouse models exposed to running exercise show increased expression of polarized aquaporin-4, which is related to glymphatic function and the accumulation of A$\upbeta$ [15]. Nevertheless, it remains unknown whether and how exercise affects the mLym system in terms of its clearance function.

To evaluate the effect of exercise on the mLym system and AD pathophysiology, we subjected 3-month-old 5xFAD transgenic mice to 3 months of voluntary running wheel exercise, until they reached 6 months of age. This exercise model was chosen for its non-stressful nature and suitability for long-term studies [16]. The two time points were chosen because, at 3 months of age, 5xFAD mice are in the initial stage of amyloid plaque accumulation, whereas by 6 months, they exhibit substantial amyloid plaque deposition. The function of the mLym system was evaluated using high-frequency ultrasound imaging (HFUS) to quantitatively monitor the drainage of intracerebroventricular (ICV) injected nanoparticles to the dCLNs as previously described [17]. In addition, we assessed the structure of mLym vessels, A$\upbeta$ deposition in the brain, and memory performance following the exercise intervention. Finally, we explored the potential underlying mechanisms of exercise-induced alterations in the mLym vasculature in lymphatic endothelial (HDLECs) and microglial (HMC3) cell lines. We focused on vascular endothelial growth factor C (VEGF-C)-VEGF receptor 3 (VEGFR3) signaling, as this pathway is the principal regulator of the mLym vasculature development [17–19].

## Materials and methods

### Mouse strains and housing

All experiments were approved by the National Cheng Kung University Institutional Animal Care and Use Committee (approval number: 109338, 111314) in accordance with the National Institutes of Health Guide for the Care and Use of Laboratory Animals. C57BL/6J wild-type (WT) mice of both sexes were obtained from the National Cheng Kung University Laboratory Animal Center, an AAALAC-accredited facility. The mice were housed in the same facility under controlled conditions, maintaining a stable temperature of 24 $\pm 1 ^{\circ }$C and a 12-h light/dark cycle (lights on at 7:00AM), with unrestricted access to food and water. Female heterozygous 5xFAD were purchased from the Jackson Laboratory and bred in-house on a C57BL/6J background. These mice carry five mutant genes associated with AD, resulting in elevated A$\upbeta$ production and an accelerated deposition of amyloid in the brain starting around two months of age.

### Running exercise procedures

In this study we have employed three different exercise paradigms. First, 3-month-old 5xFAD mice were exposed to voluntary running exercise by housing two mice per cage equipped with a running wheel. The running wheel exercise (Ex) spanned a period of three months followed a previously published protocol [16]. In general, mouse run spontaneously on running wheel for approximately 3 to 7 h with a total distance of 4 to 20 km per day. Mice of the sedentary (Sed) group were also housed two mice per cage without the running wheel.

Second, 2-month-old C57BL/6N mice were subjected to treadmill running exercise for two months. One week before the beginning of the exercise training, all mice were pre-trained on a motor-driven treadmill (Model T408E; Diagnostic & Research Instruments Co., Taoyuan, Taiwan) at the speed of 9 m/min (mild intensity exercise), 10 min/day for 5 days to familiarize the protocol. After a 2-day rest, mice underwent a running regimen during the first week, covering a speed of 16 m/min for 60 min per day, five days a week. The speed was increased 1 m/min every two weeks until it reached 19 m/min at the last week, which was considered as a moderate intensity exercise. The treadmill running exercise intervention was performed during 6:00PM to 7:00PM to conjoin with the circadian rhythm. Mice in Sed group were placed on an immobile treadmill during the training time. After finished the intervention, mouse blood in both groups was collected.

Third, 7-week-old Sprague Dawley rats were subjected to a 1-month treadmill running exercise training. Before the formal exercise training, the rats were acclimated to treadmill running at the speed of 10 m/min, 10 min/day (mild intensity) for 5 days as part of the familiarization phase. Two days later, the rats ran at a speed of 15 m/min for 60 min per day, five days a week during in the first week of the exercise phase. The speed was increased 1 m/min every week until it reached 18 m/min at the fourth week. After a 2-day rest period, the rats completed a final day of running, following the same protocol as the previous week. Blood samples were collected immediately after the exercise.

### HFUS imaging and ICV injection of nanoparticles

We used HFUS to quantitatively measure the nanoparticles draining from the lateral ventricles to the dCLNs, allowing us to evaluate the bulk flow of CSF from the brain to the dCLNs as an indicator of mLym function, as previously described [20]. Briefly, mice were placed on the stage with supine position and anesthetized with 2% isoflurane (Panion & BF Biotech Inc., Taipei City, Taiwan) at 1 L/min oxygen flow. The high-frequency transducer (40-MHz, 256-element linear array, Vevo MS500D, FUJIFILM VisualSonics Inc., Toronto, Ontario, Canada) was used to scan the neck area and image the dCLNs. Five $\upmu$l of the iron-platinum nanoparticles in poly(lactic-co-glycolic acid) nanocarriers (FePt@PLGA), manufactured as described [20], were injected into the right lateral ventricle at a flow rate of 0.5 $\upmu$l/min for 10 min. The IQ data was acquired at a frame rate of 20Hz every 5 min from the start point of injection until 60 min. For the data processing, regions of interest were examined to extract the nanoparticle signals in the dCLNs. In addition, the motion effect was removed depending on M-mode images. The extracted nanoparticle signals in the dCLNs at all pixels were then summed up and averaged to obtain signal intensity for every 5 min by using the MATLAB program (MathWorks, Natick, MA, USA).

### Quantification of the HFUS signals

The signals of HFUS FePt@PLGA nanoparticles in the dCLNs were analyzed following established protocols [20]. It has been determined that the Evans blue dye takes approximately 15 min to travel from the lateral ventricle to the dCLNs, and the peak HFUS intensity occurs around 30-35 min in mice [20]. Accordingly, these two time intervals [0-15 min for slope and 0-35 min for area under the curve (AUC)] were used to analyze metrics for mLym function. These metrics reflect the relative speed of CSF flow (slope) and the accumulated clearance of nanoparticles (AUC), respectively.

### Tissue collection and preparation

Mice were anesthetized with isoflurane (Panion & BF Biotech Inc.) and then transcardially perfused with ice-cold phosphate buffered saline (PBS). After stripping the skin and muscle around head bones, mandibles were removed. To harvest the dorsal meninges, the skullcap was clockwise cut by surgical curved scissors, beginning and ending inferior to the post-tympanic hook. Brain tissues were carefully removed from the base of skull by allowing the basal meninges remained with the skull. For immunostaining, brains and skull attached meninges were post-fixed in 4% paraformaldehyde overnight. Then, brains were dehydrated by series of graded sucrose solution, blocked in optimal cutting temperature compound (Leica Biosystems, Nussloch, Baden Wurttemberg, Germany), and sliced at a 25-$\upmu$m thickness. Coronal sections were collected in cryoprotectant (30% ethylene glycol, 20% glycerol, 50 mM PBS, pH 7.4) and stored at $-20 ^\circ$ C. For quantitative polymerase chain reaction (qPCR) analysis, samples were immediately immersed in DNA/RNA shield after harvest for further processes.

### Immunohistochemistry

The paraformaldehyde-fixed brain sections were stained for amyloid plaques. Brain sections were removed from the ethylene glycol cryoprotectant and washed three times in PBS for 10 min before incubated in PBS and 0.5% Triton X-100 containing 3% of goat serum for 1 h at room temperature. The sections were incubated in primary antibodies (mouse anti-6E10 and 4G8, each 1:200, BioLegend, San Diago, CA, USA) for 24 h at room temperature. Brain sections were washed six times with PBS and 0.5% Triton X-100 for 10 min. Then, they were incubated in diaminobenzidine tetrahydrochloride as the chromogen and nickel ammonium sulfate to intensify the reaction until the sections reached the desired staining intensity (5-10 min). Lastly, they were transferred to PBS for mounting on slides with mounting medium (Abcam, Cambridge, UK) and glass coverslips.

For immunofluorescent staining of mLym vessels, fixed whole mount-meninges were carefully stripped out from the skull and washed three times with PBS and 0.5% Triton X-100 for 10 min. Subsequently, they were incubated in PBS and 0.5% Triton X-100 containing 3% of goat serum for 1 h at room temperature before incubating in rabbit anti-lymphatic vessel endothelial hyaluronan receptor 1 (LYVE-1) antibodies (1:500, Invitrogen, Origin, UK) overnight at $4 ^\circ$C. The meninges were washed six times with PBS and 0.5% Triton X-100 for 10 min and incubated in goat Alexa Fluor 488 anti-rabbit antibodies (1:1,000, Thermo Fisher Scientific, Waltham, MA, USA,) for 2 h at room temperature in PBS and 0.5% Triton X-100. Whole mount-meninges were then washed and mounted with mounting medium (Abcam) and glass coverslips. The signals of amyloid plaques and mLym vessels were evaluated by ImageJ program version 2.1.0 (U.S. National Institutes of Health, Bethesda, Maryland, USA).

### Quantification of soluble A$\upbeta$ concentration

Soluble A$\upbeta$ concentration was determined using the commercial sandwich ELISA kit (Thermo Fisher Scientific, California, USA). The hippocampus specimens were homogenized and centrifuged at 12,000$\times$g for 15-30 min at $4 ^\circ$C. The protein concentrations were measured from the supernatant and adjusted to 0.5 mg/ml. The supernatants were diluted fivefold in Dulbecco’s PBS, then acidified to $\sim \text {pH 2.6}$, and neutralized to $\sim \text {pH 7.6}$. The soluble A$\upbeta$ levels in the supernatants were evaluated as described by the manufacturer.

### Quantification of mLym vessel diameter and coverage area

After immunofluorescent staining, the $\text {LYVE-1}^\text {+}$ signals in the dorsal and basal routes were analyzed. The dorsal route was separated into the SSS and the TS areas. The SSS mLym vessels were further divided into anterior, middle, and posterior sub-regions, whereas the TS mLym vessels were divided into proximal, middle, and distal sub-regions. To measure the diameters of mLym vessels in each sub-region, two lines were drawn perpendicular to the direction of mLym vessel flow, dividing the sub-region evenly into three parts. The diameters of vessels intersecting these lines were then quantified.

Given that the morphology of basal mLym vessels contain abundant irregular protruding capillary branches with blunt ends [18], we quantified the $\text {LYVE-1}^\text {+}$ signal coverage (% area) in bilateral boxed areas located directly adjacent to the basilar artery and situated between the vertebral artery and anterior inferior cerebellar artery. Data were averaged and analyzed for each group by using ImageJ program (U.S. National Institutes of Health).

### DCLN afferent lymphatic vessel ligation

At two and three months of age, 5xFAD mice were anesthetized using 2% isoflurane (Panion & BF Biotech Inc.) at a rate of 1 L/min of oxygen. The hair around the incision area was sterilized and removed. A longitudinal incision was made, starting from the mandible, and extending to the sternum. Using a stereomicroscope, the muscles and fascia were carefully separated from the carotid artery. The dCLNs, located near the carotid artery under the sternocleidomastoid muscle, were dissected from the surrounding tissues. The afferent lymphatic vessels of the dCLNs on the left and right sides were ligated using a thin (8-0) nylon suture (UNIK, New Taipei City, Taiwan). In sham-operated mice, only a small incision was made in the neck skin without vessel ligation. After all procedures were completed, the skin was then sutured. Following the surgery, mice were monitored and allowed to recover for a period of 4 weeks.

### Morris water maze test

The Morris water maze test was performed as described [8] with minor modifications. The whole test composed of four days of acquisition trials followed by one day of probe trial. Before beginning the test, mice were habituated in the behavior room for at least 30 min. During the acquisition period, mice underwent four trials per day for four consecutive days. The 10-cm diameter hidden platform was 1 cm below the water surface in a 120 cm diameter pool. Visual cues were placed above each quadrant of the pool. A dim light was applied in the testing room. On the first day, each mouse was placed on the platform to familiarize with the environment. Then, mouse was taken to the starting position and allowed to search the platform for 2 min. The time was stopped when mouse reached the platform. If mouse failed to find the platform within 2 min, it was gently guided to the platform. The same procedure was repeated four trails, starting from different quadrants. For the following three experimental days, the latency time was recorded for up to 2 min, and each mouse was allowed to remain on the platform for 30 sec. On the day of probe test, the platform was removed from the pool and each mouse was tested for 30 sec. All testing was conducted between 13:00PM and 18:00PM during the lights-on phase. The behavior performances were recorded by videotaping. The mean latency of four trials was calculated for each testing day. For the probe test, the percentage of time spent in target quadrant was calculated.

### A$\upbeta$ oligomer preparation

A$\upbeta$ oligomers were prepared as previously described [21]. Briefly, lyophilized A$\upbeta$ powder was dissolved in hexafluoroisopropanol at a concentration of 1 mg/mL and incubated at room temperature for 30 min. The HFIP was then allowed to evaporate overnight in open tubes within a fume hood. The resulting thin, transparent film at the bottom of the tubes was dissolved in dimethyl sulfoxide to prepare a 5 mM stock solution (0.45 mg A$\upbeta$ in 20 $\upmu$L of dimethyl sulfoxide). The stock solution was then diluted with cell culture media to achieve a final concentration of 100 $\upmu$M A$\upbeta$. Finally, the solution was incubated at $4 ^\circ$C for 24 h before cell treatment.

### Preparation of extracellular vesicles (EVs)

Blood samples were collected from the heart of rats under deep inhalation anesthesia (isoflurane) using a 23 G needle and a 20 mL syringe coated with 0.5 M EDTA solution. Then, blood was centrifuged at 4,000 x g for 10 min at $4 ^\circ$C. The supernatant was transferred to a new 15 ml tube and centrifuged at 4,000 x g for 30 min at $4 ^\circ$C to remove platelets and cell debris. Only plasma samples without hemolysis were used. The samples were then loaded onto Izon qEV10/35nm Gen 2 Columns (Izon Science Ltd., Addington, New Zealand) for size exclusion separation (35-350 nm), and each fraction was collected in 25 mL eluents. The TFF-Easy EVs concentrator (HansaBioMed Life Sciences, Tallinn, Estonia) was used to concentrate sample from 25 ml to 2 ml. Subsequently, the quantity of EVs was measured using the Nanoparticle Tracking Analysis (NTA) technique (NanoSight LM10-HS, Malvern Panalytical Ltd., Malvern, UK).

### Transmission electron microscopy

We followed a previous published procedure to perform the transmission electron microscopy [22]. In brief, the EVs were fixed in 2% PFA, placed onto formvar carbon coated electron microscopy grids, and incubated for 20 min at room temperature. Then, they were fixed in 1% glutaraldehyde for 5 min and washed with distilled water for 2 min, 8 times. The grids were exposed on 2% uranyl-oxalate for 5 min and subsequently submerged in a solution of 4% uranyl-acetate and 2% methyl cellulose for 10 min. The EVs were photographed using JEOL-2100F CS STEM at an acceleration voltage of 200kV (Tokyo, Japan).

### Western blot

The cortical brain tissue, A$\upbeta$ solutions, and extracted EVs were run on 15% polyacrylamide gels, transferred to polyvinylidene fluoride membrane (MerckMillipore/Merck KGaA, Darmstadt, Germany), probed with primary antibodies in Tris-buffered saline containing 0.1% Tween 20 overnight at $4 ^\circ$C. The primary antibodies included rabbit polyclonal anti-AQP4 (1:1,000, Proteintech, Rosemoont, USA), 6E10 mouse monoclonal antibody to A$\upbeta$ (1:5,000, BioLegend), rabbit polyclonal anti-FLOT-1 (1:1,000, Atlas Antibodies, Bromma, Stockholms Lan, Sweden), anti-HSP70 (1:1,000, GeneTex, Alton Pkwy Irvine, CA, USA), anti-CD81 (1:1,000, GeneTex), and goat polyclonal anti-clusterin (1:1,000, GeneTex). The membranes were incubated in horse radish peroxidase-conjugated secondary antibodies (1:10,000, Jackson ImmunoResearch Inc., West Grove, PA, USA) for 1 h at room temperature, washed in Tris-buffered saline with 0.1% Tween 20, and followed by incubation in ECL substrate (PerkinElmer, Waltham, Massachusetts, USA). The chemiluminescent detection was immediately performed.

### Cell culture

Human dermal lymphatic endothelial cells (HDLECs; PromoCell, Heidelberg, Germany) and human microglia cells (HMC3; American Type Culture Collection, Manassas, Virginia, USA) were cultured in endothelial cell growth medium (EGM-MV2, PromoCell) and Dulbecco’s Modified Eagle Medium (DMEM, Thermo Fisher Scientific), respectively. Both media were supplemented with 10% fetal bovine serum and 1% penicillin-streptomycin (penicillin: 10,000 units; streptomycin: 10,000 mg/mL, PSL01, Caisson Labs, East Smithfield, UT, USA). The cells were maintained in a humidified incubator at $37 ^\circ$C with 5% $\text {CO}_{\text {2}}$ and passaged four times upon reaching approximately 80% confluence before subsequent treatment.

For the A$\upbeta$ oligomer treatment study, HDLECs and HMC3 cells (50,000 cells per well) were seeded in 24-well plates and cultured in EGM-MV2 and DMEM media, respectively, for 24 h. The cells were then maintained in their respective media supplemented with 20% serum from either Ex or Sed mice or treated with 5,000 particles of EVs isolated from the serum of Ex or Sed rats for 48 h. Subsequently, the cells were washed with PBS and exposed to 0.1 $\upmu$M A$\upbeta$ oligomers in their respective media for 24 h. Following another PBS wash, the cells were harvested in sample lysis buffer (Zymo Research, CA, USA) on ice using a cell scraper for mRNA expression analysis of LYVE-1, VEGFR3, VEGF-C, and GAPDH by qPCR.

### Cytotoxicity assay

Cell Counting Kit-8 (TargetMol, MA, USA) was used to assess cytotoxicity in accordance with the manufacturer’s instructions. Briefly, 10,000 HDLECs and HMC3 were seeded in each well of a 96-well plate and cultured in EGM-MV2, and DMEM media, respectively. One day later, the culture medium was removed, and 100 $\upmu$l of A$\upbeta$ oligomer solution in medium was added for an additional 24 h. Then, 10 $\upmu$l of Cell Counting Kit-8 solution was added to each well and incubated for 1 h. The absorbance of the solution was measured at 450 nm using a microplate reader (BioTek Instruments Inc., Winooski, Vermont, USA). The experiment was performed in triplicate for each concentration.

### In-solution digestion and proteomics profiling of EVs 

For label-free quantitative proteomics profiling, 30 $\upmu$g of EV proteins were reduced by incubation with 10 mM dithiothreitol for 1 h at $29 ^\circ$C and alkylated by 55 mM iodoacetamide for 1 h at room temperature in the dark. After quenched by 55 mM dithiothreitol at $29 ^\circ$C for 45 min, proteolytic digestion was carried out using trypsin at a mass ratio of 1:50. After overnight incubation, 0.1% trifluoroacetic acid was added to stop the digestion. Peptides were then desalted with a C18 StageTip (MerckMillipore/Merck KGaA) prior to analysis. Proteomics analysis was performed using an Ultimate 3000 nanoLC system (Thermo Fisher Scientific) interfaced with an Orbitrap Fusion Lumos Tribrid mass spectrometer (Thermo Fisher Scientific). Peptide separation was carried out using a C18 Acclaim PepMap column packed with 2 $\upmu$m particles with a pore of 100 Å (Thermo Scientific). Mobile phase A was 0.1% formic acid in water, and mobile phase B was composed of 100% acetonitrile with 0.1% formic acid. A segmented gradient in 90 min from 2% to 35% solvent B at a flow rate of 300 nl/min and a column temperature of $35^\circ$C were used. Data were collected under data-dependent acquisition mode, where the most intense ions in 3 sec with charge states 2-7 were isolated using standard DDA acquisition with HCD fragmentation and high-resolution Orbitrap detection. MS1 and MS/MS spectra were acquired using the Orbitrap analyzer at resolving powers of 120,000 and 60,000, respectively. The resulting raw spectra were analyzed using Mascot within Proteome Discoverer software (version 2.3; Matrix Science, London, UK), searching against a rat protein sequence database. Label-free quantification was employed. The search parameters included tryptic digestion allowing up to two missed cleavages, a precursor ion mass tolerance of 10 ppm, and a fragment ion tolerance of 0.02 Da. Carbamidomethylation of cysteine was defined as a fixed modification, while oxidation of methionine and deamidation on asparagine and glutamine were treated as variable modifications. Peptide identifications were filtered using a false discovery rate (FDR) threshold below 1%. Proteomics data processing was carried out using Perseus software (version 2.0.5.0), where log2 transformation was applied to normalize the distribution and to enable downstream statistical analysis. Missing values were substituted via an imputation algorithm to minimize data bias. For functional annotation, EV proteins were subjected to Gene Ontology (GO) and Kyoto Encyclopedia of Genes and Genomes (KEGG) pathway enrichment analysis using the DAVID bioinformatics platform. Group-wise comparisons were performed using an unpaired Student’s t-test on log-transformed protein abundance values, and statistical significance was defined as a *p*-value below 0.05.

### RNA extraction and qPCR measurement

Total RNA was extracted from brain tissues or cultured cells using the Quick-RNA Miniprep Plus kit (Zymo Research) according to the manufacturer’s instructions. RNA samples were then reverse transcribed into cDNA (500 ng) using the $\text {PrimeScript}^{\text {TM}}$ RT Master Mix (Takara Bio, Kusatsu, Japan). For qPCR, the cDNA templates were amplified with SYBR Green PCR Master Mix (Takara Bio) on a StepOnePlus Real-Time PCR System (Applied Biosystems, Thermo Fisher Scientific). The expression levels of target genes were normalized to Gapdh mRNA levels. The specific primer sequences for each gene (MB Mission Biotech, Taipei, Taiwan) are listed in Table 1.

**Table 1:** Primer sequences for qPCR

Gene	Forward primer sequence $5' \rightarrow 3'$	Reverse primer sequence $5' \rightarrow 3'$
LYVE-1	TGGTGTTACTCCTCGCCTCT	TTCTGCGCTGACTCTACCTG
VEGFR3	CTCTCCAACTTCTTGCGTGTC	GCTTCCAGGTCTCCTCCTATC
VEGF-C	TCTGTGTCCAGCGTAGATGAG	GTCCCCTGTCCTGGTATTGAG
CD9 (mouse)	CTGTGGCATAGCTGGTCCTTTG	AGACCTCACTGATGGCTTCAGG
CD9 (human)	CTGGGACTGTTCTTCGGCTT	GATGGCTTTCAGCGTTTCCC
GAPDH (mouse)	ACCCAGAAGACTGTGGATGG	CACATTGGGGGTAGGAACAC
GAPDH (human)	GAGTCAACGGATTTGGTCGT	TTGATTTTGGAGGGATCTCG

### Statistical Analysis

Data are presented as $\text {mean} \pm \text {standard deviation}$. Since all datasets followed a normal distribution, we used the unpaired, two-tailed Student’s t-test to compare means between two groups. The association between amyloid plaque load and slope/AUC of the HFUS signals were evaluated using the Pearson correlation. Results of mLym gene expression among different groups were analyzed by one-way analysis of variance (ANOVA) followed by Sidak’s multiple comparison tests. The two-way ANOVA with repeated measures was used to analyze the two main effects and possible interaction for mLym function and Morris water maze learning curve. Statistical significance was considered at $\textit{p} < 0.05$ and all statistical analysis was performed by Prism program (Version 10.1.1).

## Results

### The function of mLym system decreases with age in 5xFAD mice

**Fig. 1:**
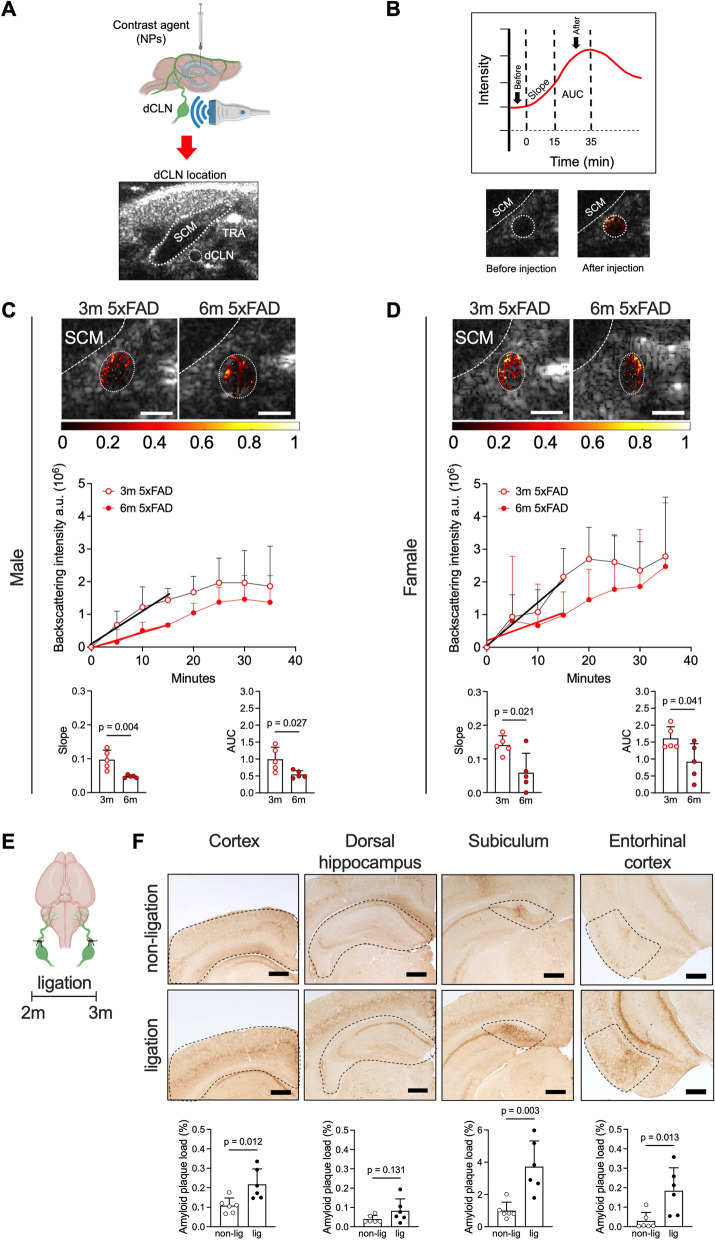
Age-dependent decline in meningeal lymphatic function in 5xFAD mice. **A** Upper Panel: Schematic illustration of real-time monitoring of CSF flow using high-frequency ultrasound (HFUS) imaging enhanced with FePt@PLGA nanoparticle contrast agents. The ultrasound transducer is positioned to scan the deep cervical lymph nodes (dCLNs) while nanoparticles are administered into the lateral ventricles. Lower Panel: Representative HFUS image displaying the dCLN, delineated by a dashed circle, indicating the region of interest for CSF flow assessment. SCM: sternocleidomastoid muscle; TRA: trachea. **B** Upper Panel: Schematic illustration of HFUS signal intensity traces of the dCLN region captured before and after intracerebroventricular (ICV) injection of FePt@PLGA nanoparticles. The slope and area under the curve (AUC) of these traces serve as quantitative metrics for meningeal lymphatic function, reflecting the rate and volume of CSF flow from the lateral ventricles to the dCLNs, respectively. Lower Panels: Corresponding HFUS images of the dCLN region taken before and after nanoparticle injection. **C, D** Upper Panels: Representative HFUS images with nanoparticle signal mapping at the dCLN (dashed circle) of 3- and 6-month-old 5xFAD male **(C)** and female **(D)** mice at 35 min post-ICV injection. Scale bar = 1 mm. The color bar represents the normalized intensity of backscattering signals. Middle Panels: Averaged HFUS signal intensity traces from 0 to 35 min and averaged slope from 0 to 15 min (straight line). Lower Panels: Quantitative results of slope and AUC. N = 5. Unpaired two-tailed Student’s t-test. **E** Schematic diagram depicting the surgical ligation of afferent lymphatic vessels and the duration of the ligation procedure. **F** Representative micrographs of A$\upbeta$ immunostaining in the cortex, dorsal hippocampus, subiculum, and entorhinal cortex regions (outlined by dashed lines) of 3-month-old 5xFAD mice subjected to either one month of afferent lymphatic vessel ligation or sham (non-ligation) treatment. Scale bar = 500 $\upmu$m. Quantitative results are shown on their respective lower panels. N = 6. Unpaired two-tailed Student’s t-test

Meningeal lymphatic function was assessed using HFUS with a FePt@PLGA nanoparticle contrast agent in 5xFAD mice at 3 and 6 months of age, corresponding to established stages of A$\upbeta$ pathology progression in this transgenic model. Amyloid plaque deposits typically begin around 2-3 months, with more extensive plaque deposition becoming evident by 6 months in regions including the cortex, dorsal hippocampus, subiculum, and entorhinal cortex (Supplementary Fig. 1). No sex-dependent differences were observed at these time points (Supplementary Fig. 1). HFUS imaging of the dCLNs enabled quantitative monitoring of FePt@PLGA nanoparticle drainage from the lateral ventricle to the dCLNs (Fig. 1A). The HFUS signal has been validated to specifically originate from mLym drainage of FePt@PLGA nanoparticles, as demonstrated by the reduction in HFUS signal intensity at the dCLNs following photoconversion-mediated ablation of mLym vessels near the confluence of sinuses [20]. It has been shown that ICV injection of Evans blue dye reaches the dCLNs within approximately 15 min, while FePt@PLGA nanoparticles yield maximal HFUS signal intensities at around 35 min post-injection in mice [20]. Based on these kinetics, the 0-15 min interval was used to calculate CSF flow rate (slope), while the 0-35 min window was used to assess cumulative nanoparticle clearance via area under the curve (AUC) analysis [20] (Fig. 1B). Compared to 3-month-old 5xFAD mice, 6-month-old 5xFAD mice exhibited a significant decrease in both the slopes and the AUC of HFUS signal intensities in both sexes (Fig. 1C, D). In contrast, no such age-dependent alterations were observed in age-matched WT controls of either sex (Supplementary Fig. 2).

To demonstrate the importance of the mLym system in A$\upbeta$ clearance, we surgically ligated the left and right afferent lymphatic vessels at the points just before they enter dCLNs in 2-month-old 5xFAD mice (Fig. 1E). One month after ligation, the amyloid plaque loads in the cortex, subiculum and entorhinal cortex were increased, although this increase did not reach statistical significance in the dorsal hippocampus (Fig. 1F). These results support the notion that the age-associated deterioration of the mLym drainage contributes to accelerated amyloid plaque accumulation in the brain.

### Exercise enhances the function of mLym system and A$\upbeta$ clearance in 5xFAD mice

**Fig. 2:**
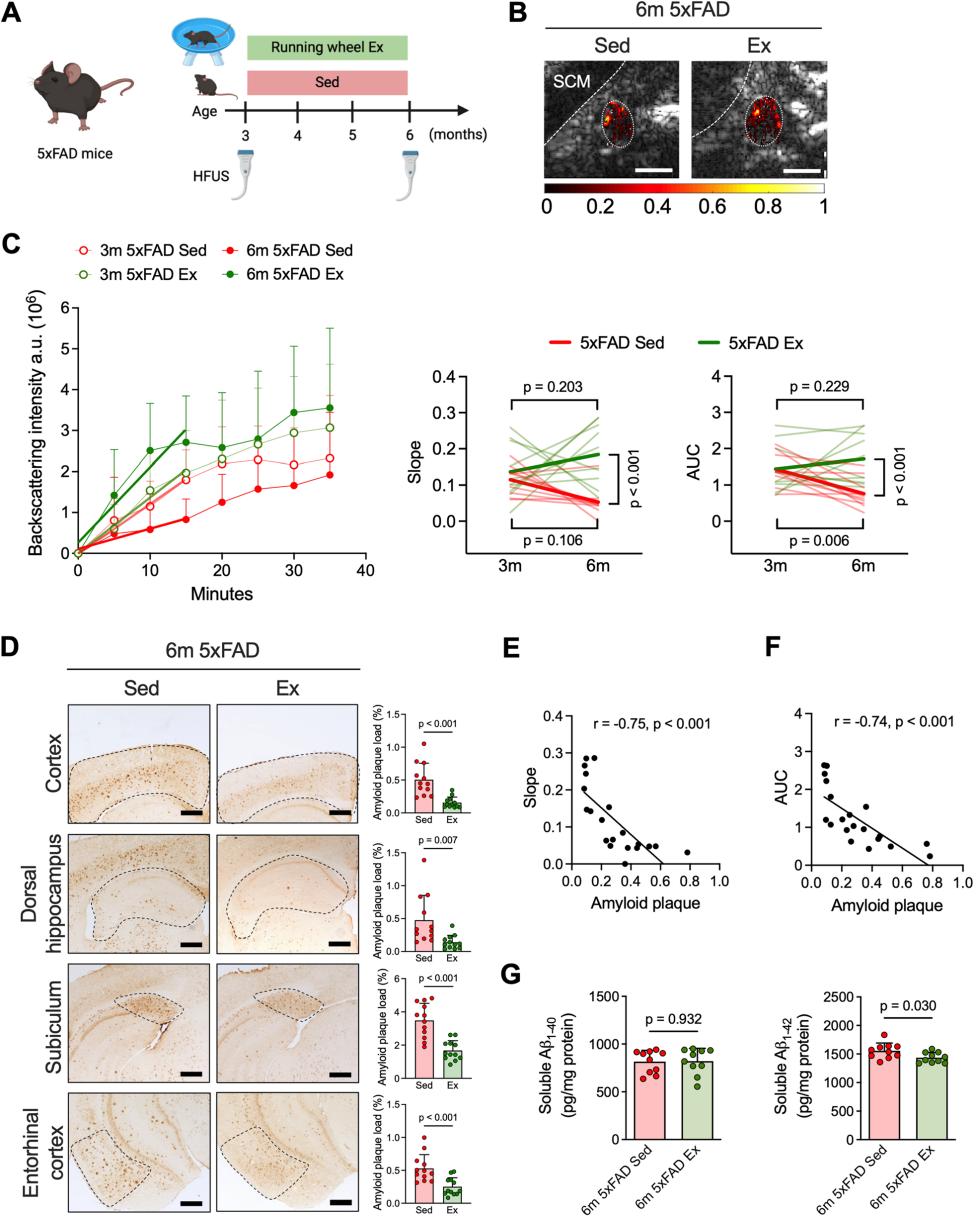
Exercise enhances meningeal lymphatic function and reduces amyloid plaque accumulation in 5xFAD mice. **A** Timeline outlining the assessment of meningeal lymphatic function via high-frequency ultrasound (HFUS) imaging conducted before and after a 3-month period of voluntary running exercise (Ex). Sed: sedentary. **B** Visualization of HFUS nanoparticle signals mapping within the deep cervical lymph node region (dashed circle) in 6-month-old 5xFAD mice, captured 35 min post-intracerebroventricular injection. SCM: sternocleidomastoid muscle. Scale bar = 1 mm. The accompanying color bar denotes the normalized intensity of backscattered signals. **C** Averaged HFUS signal intensity traces from 0 to 35 min and corresponding slopes from 0 to 15 min in 3- and 6-month-old 5xFAD mice, with or without Ex intervention. Right panels display paired line plots illustrating individual mouse data, with thicker lines representing group means. P-values at the top indicate changes within the Ex groups, while those at the bottom refer to the Sed groups. N = 10. Repeated measures two-way ANOVA. **D** Representative micrographs showing A$\upbeta$ immunostaining in the cortex, dorsal hippocampus, subiculum, and entorhinal cortex regions (outlined by dashed lines) of 6-month-old 5xFAD mice, comparing Ex and Sed groups. Scale bar = 500 $\upmu$m. Quantitative analyses are presented in the right panels. N = 12. Unpaired two-tailed Student’s t-test. **E** Correlation between the slope of HFUS signal traces (0 to 15 min) and amyloid plaque load in 6-month-old 5xFAD mice, with or without Ex treatment. **F** Correlation between the area under the curve (AUC) of HFUS signal traces (0 to 35 min) and amyloid plaque load in the same cohort of mice. **G** Levels of soluble $\text {A}\upbeta _{\text {1-40}}$ and $\text {A}\upbeta _{\text {1-42}}$ in the hippocampus of 6-month-old 5xFAD mice, with or without Ex treatment. N = 10. Unpaired two-tailed Student’s t-test

To examine how exercise affects the mLym system, we exposed 3-month-old 5xFAD mice to voluntary running exercise (Ex) for a duration of 3 months and monitored the function of mLym system before and after the 3-month exercise regimen (Fig. 2A). HFUS signal mapping revealed that 6-month-old Ex mice exhibited greater nanoparticle intensity at the dCLN in comparison to the age-matched Sed mice (Fig. 2B). Furthermore, the HFUS results showed that age-related deterioration in mLym function (i.e., slope and AUC) of 5xFAD mice was alleviated by 3 months of Ex (Fig. 2C). Since the beneficial effect of Ex was evident in both male and female 5xFAD mice (Supplementary Fig. 3), we combined the results from both sexes.

Immunostaining analyses revealed decreased amyloid plaque loads in the four selected brain regions of the Ex group compared to the age-matched Sed group (Fig. 2D). When plotting the slopes and AUCs of the HFUS signals against amyloid plaque loads, robust negative correlations were observed in the cortex (Fig. 2E, F), as well as the other three brain regions examined (Supplementary Fig. 4). Compared to Sed controls, the 6-month-old Ex group had significantly reduced levels of soluble $\text {A}\upbeta _{\text {1-42}}$ but not soluble $\text {A}\upbeta _{\text {1-40}}$ in the hippocampus (Fig. 2G). These findings suggest that exercise can enhance the function of the mLym system, resulting in improved A$\upbeta$ clearance and lower amyloid plaque load in the brain.

**Fig. 3:**
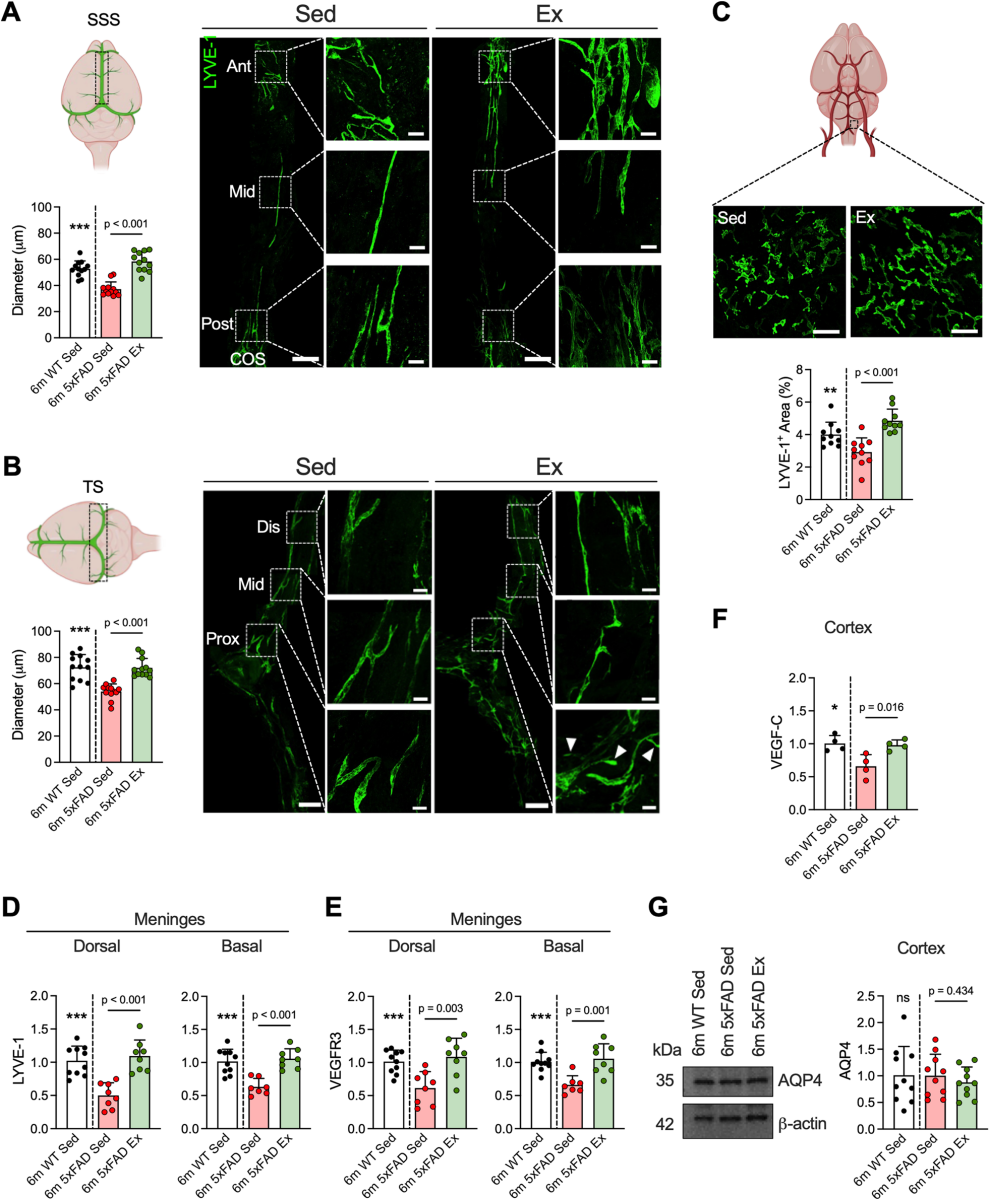
Exercise enhances lymphangiogenesis and the VEGF-C/VEGFR3 signaling pathway. **A,B** Cartoon diagrams display the mLym vessels along the superior sagittal sinus (SSS, black dash line box) **(A)** and transverse sinus (TS, black dash line box) **(B)**. Representative LYVE-1 immunofluorescent micrographs along the SSS and TS of 5xFAD Sed and Ex mice at 6 months of age. Enlarged views of the anterior (Ant), middle (Mid), and posterior (Post) sub-regions (white boxed areas) for the SSS **(A)** and the proximal (Prox), middle (Mid), and distal (Dis) sub-regions (white boxed areas) for the TS **(B)** are shown on their respective right panels. COS: confluence of sinus. The bars are 500 $\upmu$m for full images and 100 $\upmu$m for enlarged images. Quantitative results of averaged diameter measurements for three sub-regions are shown in the lower left panel. N = 12. Unpaired two-tailed Student’s t-test. **C** A cartoon diagram illustrates the designated area for quantifying the basal route of mLym vessels. The panels below display representative LYVE-1 immunofluorescent micrographs in the boxed region. Scale bar = 100 $\upmu$m. Quantitative results of $\text {LYVE-1}^\text {+}$ area (%) of basal mLym vessels. N = 10. Unpaired two-tailed Student’s t-test. **D-F** Quantitative results of gene expression for LYVE-1 **(D)** and VEGFR3 **(E)** in the dorsal and basal parts of meninges (n = 8) and VEGF-C **(F)** in the cortex (n = 4). Unpaired two-tailed Student’s t-test. **G** Immunoblot and quantitative results of AQP4 protein expression in the cortex. N = 10. Unpaired two-tailed Student’s t-test. In all quantitative analyses, the age-matched wild-type (WT) littermate group was used as a reference to demonstrate the effect of the 5xFAD transgene (WT Sed vs. 5xFAD Sed; ns = not significant, * $\textit{p} < 0.05$, ** $\textit{p} < 0.01$, *** $\textit{p} < 0.001$, Unpaired two-tailed Student’s t-test), and it was not included in the statistical analyses evaluating the exercise effect (5xFAD Sed vs. 5xFAD Ex)

### Exercise prevents mLym vessel deterioration in 5xFAD mice

The effect of Ex on the morphology of mLym vessels was determined in both the dorsal and basal routes. In the dorsal route, the diameters of $\text {LYVE-1}^\text {+}$ vessels along the SSS (Fig. 3A) and TS (Fig. 3B) were determined by averaging measurements from three sub-regions within each respective area. In 6-month-old 5xFAD Sed mice, the diameters of $\text {LYVE-1}^\text {+}$ vessels along the SSS and TS were smaller compared to age-matched WT Sed mice (Fig. 3A, B). Three months of exercise rescued these morphological changes (Fig. 3A, B), with no sex differences (Supplementary Fig. 5B, C). Additionally, we observed small $\text {LYVE-1}^\text {+}$ vessels branching out from the main trunk in the TS mLym vessels of the Ex mice (Fig. 3B, white arrowheads in the Ex proximal sub-region), indicating that Ex promotes lymphangiogenesis. In the basal route, the $\text {LYVE-1}^\text {+}$ signals in 6-month-old 5xFAD Sed mice were lower than those in age-matched WT Sed mice (Fig. 3C). These reduced levels could also be rescued by Ex in both male and female mice (Fig. 3C, 5xFAD Sed vs. 5xFAD Ex, Supplementary Fig. 5D).

The exercise-induced increases in the coverage and diameters of mLym vessels suggest an enhancement in lymphangiogenesis, a process that is critically regulated by VEGF-C/VEGFR3 signaling, with immune cells like microglia being the primary source of VEGF-C within the central nervous system [23–25]. Thus, we quantified the expression levels of LYVE-1 and VEGFR3 in the meninges and measured the levels of VEGF-C in cortical tissue. Compared to age-matched WT Sed mice, 6-month-old 5xFAD Sed mice exhibited reduced expression levels of LYVE-1 and VEGFR3 in both the dorsal and basal meninges, as well as decreased VEGF-C expression in the cortex (Fig. 3D-F). 5xFAD mice that received Ex had higher expression levels for all three genes than those of the age-matched 5xFAD Sed group (Fig. 3D-F). No sex-specific differences were noted in any of the expression results (Supplementary Fig. 6).

Since CNS lymphatic clearance links the glymphatic and mLym pathways, and aquaporin-4 (AQP4) is a key astrocytic water channel for glymphatic CSF-ISF exchange [26], we assessed whether Ex affects cortical AQP4 expression in 5xFAD mice. Western blots showed no significant differences in total AQP4 levels among WT, 5xFAD Sed, and 5xFAD Ex mice (Fig. 3G), consistent in both sexes (Supplementary Fig. 7).

### Exercise enhances learning and memory performance in 5xFAD mice

**Fig. 4:**
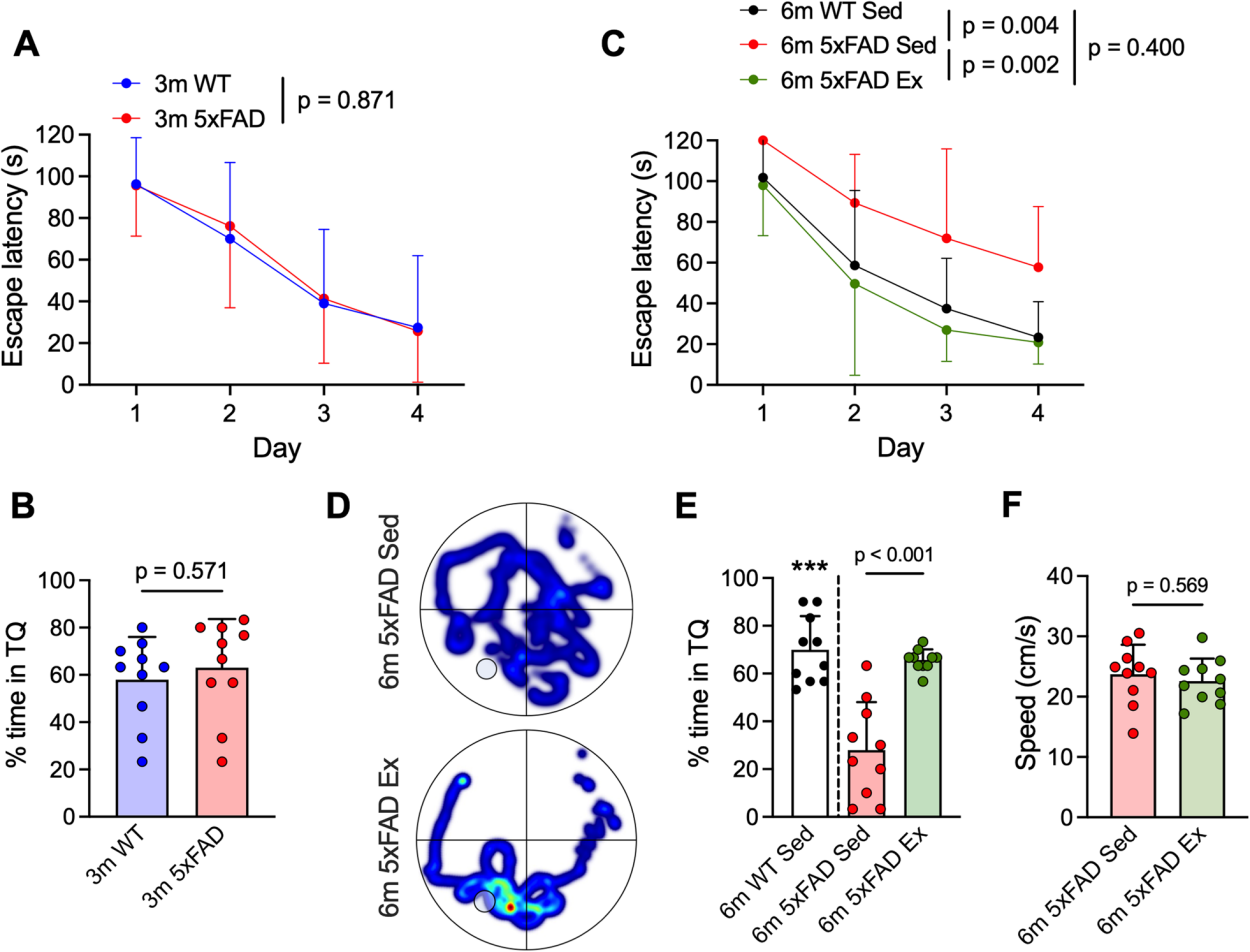
Exercise improves learning and memory performances in 5xFAD mice. **A **Trends of escape latency of WT and 5xFAD mice at 3 months of age. Repeated measures two-way ANOVA. **B** Quantitative result of percent time in target quadrant. N = 10. Unpaired two-tailed Student’s t-test. **C** Trends of escape latency of 6-month-old WT and 5xFAD mice with or without Ex. Repeated measures two-way ANOVA. **D** Heat map representations of swim path of 6-month-old 5xFAD Sed and Ex groups. Little circle in the lower left quadrant marks the location of platform. **E** Quantitative result of percent time in target quadrant. N = 10. Unpaired two-tailed Student’s t-test. **F** Quantitative result of swim speed. N = 10. Unpaired two-tailed Student’s t-test. The aged-matched wild-type (WT) littermate group was used as a reference to demonstrate the effect of the 5xFAD transgene (WT Sed vs. 5xFAD Sed; *** $\textit{p} < 0.001$, Unpaired two-tailed Student’s t-test), and it was not included in the statistical analyses evaluating the exercise effect (5xFAD Sed vs. 5xFAD Ex)

The spatial learning and memory abilities of the mice were assessed before and after the 3-month exercise regimen using the Morris water maze. The results showed that there was no difference in learning (measured by escape latency) (Fig. 4A) and memory (assessed by time spent in the target quadrant) (Fig. 4B) abilities between WT and 5xFAD mice at 3 months of age. Both male and female mice yielded similar results (Supplementary Fig. 8A).

At 6 months of age, the 5xFAD Sed group showed impaired learning performance compared to the WT Sed group, a deficit that was prevented by the 3-month Ex intervention (Fig. 4C). Likewise, 5xFAD Sed mice exhibited reduced memory performance compared to age-matched WT Sed mice, which was restored by the 3-month exercise intervention (Fig. 4D, E). Swim speed was unaffected by Ex in 6-month-old 5xFAD mice, with no sex differences observed (Fig. 4F, Supplementary Fig. 8B). Notably, the beneficial effect of Ex was more pronounced in male mice than in female counterparts (Supplementary Fig. 8B).

### Blockage of mLym pathway abolishes exercise-induced benefits on A$\upbeta$ clearance and learning and memory performance in 5xFAD mice

**Fig. 5:**
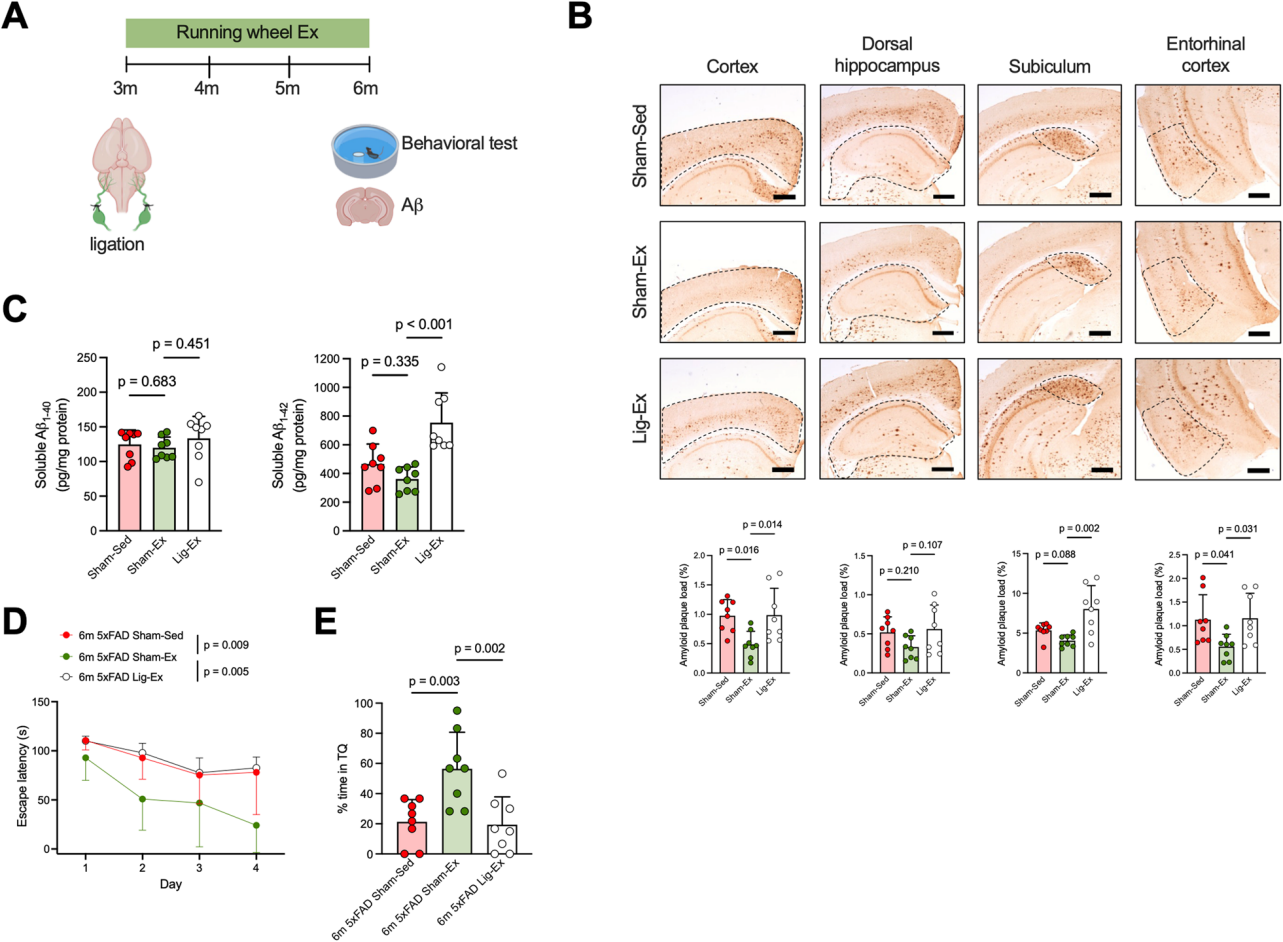
Lymphatic vessel blocking suppresses exercise-related benefits. **A **Timeline outlining the surgical ligation of afferent lymphatic vessels prior entering dCLNs, the behavioral and amyloid-$\upbeta$ accumulation assessment. Ex = running exercise. **B** Representative micrographs showing A$\upbeta$ immunostaining in the cortex, dorsal hippocampus, subiculum, and entorhinal cortex regions (outlined by dashed lines) of sham-sedentary, sham-exercise, and ligation-exercise 5xFAD mice at 6 months of age. Scale bar = 500 $\upmu$m. Quantitative analyses are presented in the lower panels. N = 8. One-way ANOVA. **C** Levels of soluble $\text {A}\upbeta _{\text {1-40}}$ (left) and $\text {A}\upbeta _{\text {1-42}}$ (right) in the hippocampus of 6-month-old 5xFAD mice, with or without ligation and Ex treatment. N = 8. One-way ANOVA. **D** Trends of escape latency of 6-month-old 5xFAD mice with or without ligation and Ex treatment. Repeated measures two-way ANOVA. **E** Quantitative result of percent time in target quadrant. N = 8. One-way ANOVA

To assess whether the mLym pathway mediates the effects of exercise on A$\upbeta$ clearance and cognitive improvement, we bilaterally ligated the afferent lymphatic vessels entering the dCLNs in 3-month-old 5xFAD mice before initiating the 3-month exercise intervention (Fig. 5A). Immunostaining showed that lymphatic ligation significantly abolished the exercise-induced decrease in amyloid plaque burden, particularly in the cortex, subiculum, and entorhinal cortex, while changes in the dorsal hippocampus did not reach statistical significance (Fig. 5B). The exercise-induced reduction in soluble $\text {A}\upbeta _{\text {1-42}}$ levels was also abolished when lymphatic drainage was blocked, whereas soluble $\text {A}\upbeta _{\text {1-40}}$ levels in the hippocampus were unchanged (Fig. 5C). Furthermore, the exercise-induced improvement in learning and memory were substantially diminished following lymphatic ligation (Fig. 5D, E).

### Serum and EVs from exercised animals protect lymphatic endothelial cells from A$\upbeta$ oligomer-induced damage

**Fig. 6:**
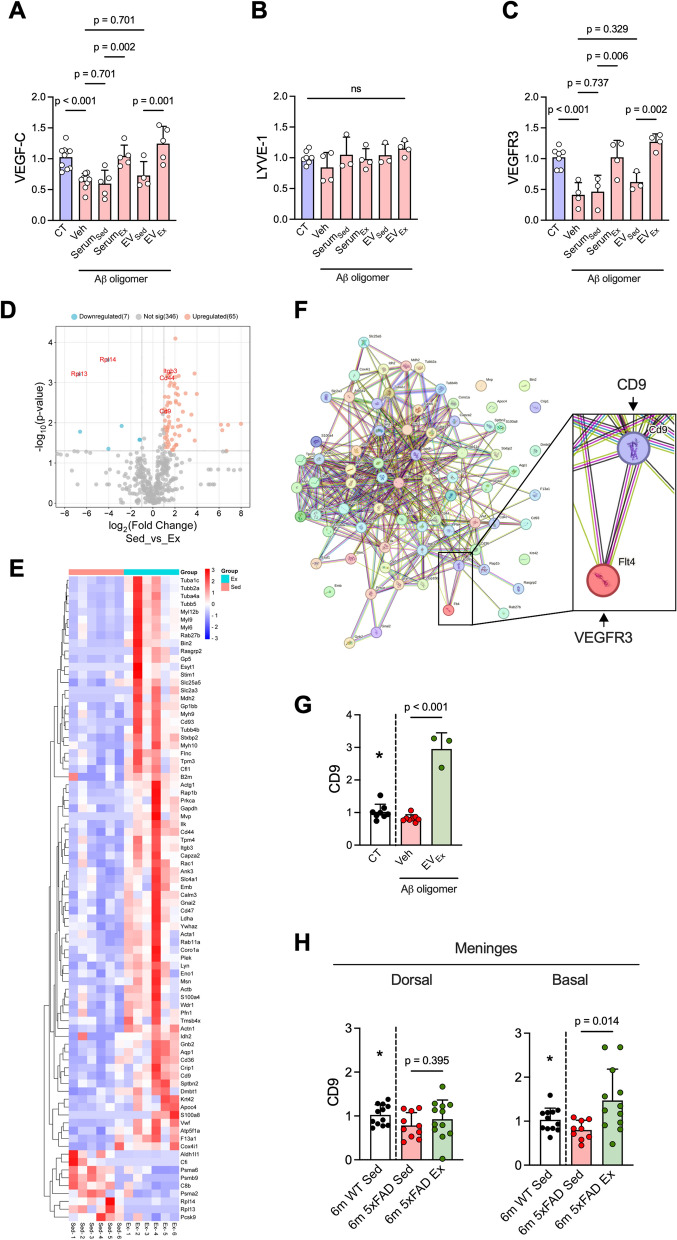
Serum and extracellular vesicles from exercised animals inhibit A$\upbeta$ oligomer-induced downregulation of lymphatic vessel-related genes. **A-C** Quantitative results of lymphatic vessel-related gene expression in response to A$\upbeta$ oligomer and treatments with serum or extracellular vesicles (EVs) derived from sedentary ($\text {Serum}_{\text {Sed}}$ and $\text {EV}_{\text {Sed}}$) or exercised ($\text {Serum}_{\text {Ex}}$ and $\text {EV}_{\text {Ex}}$) animals. **A** Levels of VEGF-C in microglial HMC3 cells. **B** Levels of LYVE-1 in human dermal lymphatic endothelial cells (HDLECs). **C** Levels of VEGFR3 in HDLECs. CT: control cells without any treatment. Veh: cells with A$\upbeta$ oligomer treatment only. N = 3-10. One-way ANOVAs. **D** Volcano plot. Differential gene expression analysis of EVs from exercised ($\text {EV}_{\text {Ex}}$) versus sedentary ($\text {EV}_{\text {Sed}}$) animals. Red dots denote upregulated genes, and blue dots indicate downregulated genes. **E** Heat Map. Visualization of gene expression profiles in EVs from $\text {EV}_{\text {Ex}}$ and $\text {EV}_{\text {Sed}}$ groups. Expression intensities are color-coded from red (high expression) to blue (low expression). N = 6. **F** STRING network diagram. Protein-protein interaction network derived from 82 differentially expressed genes in EVs. The black box highlights interactions between EV-associated genes and VEGFR3. **G** Quantitative results of CD9 levels in HDLECs treated with A$\upbeta$ oligomers and $\text {EV}_{\text {Ex}}$. **H** Quantitative results of CD9 gene expression in the dorsal and basal parts of the meninges. CT: control (untreated); Veh: A$\upbeta$ oligomer treatment only. N = 9-12. Unpaired two-tailed Student’s t-test

To investigate the protective effects of Ex on mLym vessels, HDLECs and HMC3 cells were cultured for 24 h in media supplemented with 20% serum from Ex ($\text {Serum}_{\text {Ex}}$) or Sed ($\text {Serum}_{\text {Sed}}$) mice, followed by treatment with A$\upbeta$ oligomers. Dose-response analysis showed that A$\upbeta$ oligomers did not affect HDLEC viability at concentrations up to 0.1 $\upmu$M, whereas HMC3 viability was unaffected up to 1 $\upmu$M (Supplementary Fig. 9A-C). Moreover, at sub-lethal concentrations, A$\upbeta$ oligomers induced a dose-dependent reduction in VEGFR3 expression in HDLECs (Supplementary Fig. 9D). Based on this, we selected 0.1 $\upmu$M for subsequent experiments, as it was the highest effective non-toxic concentration and aligned with A$\upbeta$ levels observed in human AD disease [27]. At this concentration, A$\upbeta$ oligomers significantly reduced VEGF-C expression in HMC3 cells and VEGFR3 expression in HDLECs (Fig. 6A, C, Veh vs. CT, Supplementary Fig. 9D), without altering LYVE-1 levels in HDLECs (Fig. 6B). Importantly, pre-treatment with $\text {Serum}_{\text {Ex}}$, but not $\text {Serum}_{\text {Sed}}$, prevented the A$\upbeta$ oligomer-induced reduction in VEGF-C and VEGFR3.

To identify exercise-induced circulating factors, we isolated EVs from rat plasma after one month of moderate-intensity treadmill running ($\text {EV}_{\text {Ex}}$) or no exercise ($\text {EV}_{\text {Sed}}$). NTA results revealed that $\text {EV}_{\text {Ex}}$ and $\text {EV}_{\text {Sed}}$ samples exhibited comparable particle concentrations, size distributions, and mean diameters (Supplementary Fig. 10A-E). Transmission electron microscopy showed that the EVs exhibited either a round or oval-shaped morphology, with diameters ranging from 100 to 200 nm (Supplementary Fig. 10F). Western blot analysis confirmed the presence of EV markers FLOT-1, HSP70, Clusterin, and CD81 in the isolated EVs [28] (Supplementary Fig. 10G).

Similar to the effects observed with serum, pre-treatment with $\text {EV}_{\text {Ex}}$, but not $\text {EV}_{\text {Sed}}$ prevented the A$\upbeta$ oligomer-induced reduction in VEGF-C and VEGFR3 expression (Fig. 6A, C). Neither $\text {EV}_{\text {Ex}}$ nor $\text {EV}_{\text {Sed}}$ affected LYVE-1 expression (Fig. 6B). These results indicate that both serum and EVs derived from exercised animals can protect lymphatic endothelial cells from A$\upbeta$ oligomer-induced damage.

To elucidate the molecular mediators of this protective effects, we performed quantitative proteomic profiling of $\text {EV}_{\text {Ex}}$ and $\text {EV}_{\text {Sed}}$. Among 418 identified proteins, differential expression analysis revealed 65 upregulated and 7 downregulated proteins in $\text {EV}_{\text {Ex}}$ (Fig. 6D). In total, 82 proteins with significant expression changes were visualized via hierarchical clustering (Fig. 6E). STRING-based protein-protein interaction and pathway enrichment analysis of these 82 proteins, together with VEGFR3 (FLT4), identified a network centered on CD9, a tetraspanin involved in membrane organization, signaling, and vesicular trafficking (Fig. 6F) [29].

To assess the relevance of CD9, we treated HDLECs with A$\upbeta$ oligomers in the presence or absence of $\text {EV}_{\text {Ex}}$. A$\upbeta$ oligomers alone reduced CD9 expression (Fig. 6G, Veh vs. CT), whereas $\text {EV}_{\text {Ex}}$ significantly restored its levels (Fig. 6G, Veh vs. $\text {EV}_{\text {Ex}}$). Consistently, 6-month-old 5xFAD Sed mice showed reduced CD9 expression in both dorsal and basal meninges compared to age-matched WT Sed controls. Exercise increased CD9 expression in 5xFAD mice, particularly in the basal meninges (Fig. 6H). Together, these findings suggest that exercise protects lymphatic endothelial cells from A$\upbeta$ oligomer-induced damage by promoting the expression of lymphatic vessel-related genes, including CD9.

## Discussion

Using real-time HFUS imaging with FePt@PLGA nanoparticles contrast, we monitored CSF drainage to dCLNs in 5xFAD mice. In sedentary 5xFAD mice, we observed an age-dependent decline in mLym function, which correlated with increasing A$\upbeta$ accumulation and memory deficits. This decline was accompanied by reduced expression of lymphatic endothelial markers LYVE-1 and VEGFR3 in the meninges and decreased brain VEGF-C levels in older 5xFAD animals. Importantly, surgical ligation of mLym vessels in young 5xFAD mice accelerated brain amyloid accumulation, highlighting the importance of mLym drainage in A$\upbeta$ clearance. These findings support previous studies linking impaired mLym function to exacerbated A$\upbeta$ pathology in AD models [8, 30].

In contrast, a long-term voluntary wheel-running regimen exerted robust protective effects. Exercised 5xFAD mice showed improved mLym vessel morphology and increased CSF drainage to dCLNs. This was associated with reduced parenchymal A$\upbeta$ burden and improved cognitive performance. At the molecular level, exercise restored LYVE-1 and VEGFR3 expression in the mLym tissue and increased VEGF-C levels in the brain. *In vitro*, A$\upbeta$ oligomers suppressed VEGFR3 expression in HDLECs, but co-treatment with $\text {Serum}_{\text {Ex}}$ or $\text {EV}_{\text {Ex}}$ rescued this suppression. Although exercise did not change the total number of circulating EVs, it altered their composition enabling them to counteract A$\upbeta$-induced VEGFR3 downregulation. Altogether, these findings demonstrate that voluntary physical activity preserves mLym function, reducing A$\upbeta$ accumulation, and supports cognitive resilience in 5xFAD mice.

Physical exercise has been linked to delayed onset of AD pathology, in part due to enhancement of the glymphatic system [11, 14, 31, 32]. Long-term exercise increases CSF influx and clearance, partially through upregulation of polarized aquaporin-4 [15, 32]. Here, we focused on the downstream mLym system and demonstrated that blocking it worsened amyloid deposition, whereas sustained voluntary running promotes CSF drainage, reduces brain A$\upbeta$ load, and improves memory in 5xFAD mice. Aligned with our findings, a recent study utilizing noninvasive magnetic resonance imaging in healthy human demonstrated that long-term physical exercise significantly increases both the size and CSF flow of mLym vessels, underscoring potential role in promoting brain waste clearance [33]. Collectively, these observations support a model in which exercise protects against AD at least partially through structural and functional support of the meningeal lymphatic vasculature. Our observation that voluntary exercise decreases A$\upbeta$ burden and enhances cognition in 5xFAD mice aligns with numerous prior reports [34–36], yet some studies have not demonstrated comparable effects. Svensson et al. [37], using a similar running paradigm, found no change in CSF soluble A$\upbeta$ or Thioflavin S-positive plaques when exercise was extended to 8 months of age. Because amyloid pathology and neurodegeneration are markedly more advanced at this stage, any early benefits of exercise may have been attenuated or no longer detectable. Furthermore, methodological differences may contribute to these discrepant outcomes. We assessed amyloid pathology using immunohistochemistry, which detects plaques across a broad range of maturation states, whereas Thioflavin S selectively labels $\upbeta$-sheet-rich mature plaques [38]. This distinction may lead to underestimation of exercise effects on early or diffuse plaque forms in studies relying solely on Thioflavin S staining. In addition, behavioral endpoints differed across studies. Svensson et al. [37] focused primarily on anxiety- and motor-related tasks, which depend on brain regions with relatively mild amyloid pathology (e.g., ventral hippocampus, nigrostriatal pathway, cerebellum). In contrast, our work assessed hippocampal-dependent learning and memory, functions tied to the dorsal hippocampus, a region with substantial amyloid deposition in 5xFAD mice. These differences in both pathological burden and behavioral sensitivity may account for the variability in exercise-related outcomes.

Although female 5xFAD mice frequently develop earlier and more severe A$\upbeta$ pathology than males [39, 40], our study found no statistically significant sex differences in plaque load across the examined brain regions at 3-6 months of age. This likely reflects the early disease stage analyzed, characterized by emerging amyloid deposition and high inter-animal variability [40, 41]. Similarly, structural and functional measures of the mLym system showed no significant sex differences. Exercise tended to enhance mLym function more in males than females, although the difference was not significant, aligning with previous reports that physical activity improves glymphatic and lymphatic clearance broadly, without robust sex-specific effects in early-stage 5xFAD mice [42]. These findings suggest that sex differences in amyloid pathology and mLym function may become more pronounced at later stages of disease, highlighting the importance of longitudinal studies to fully characterize sex-dependent responses in AD mouse models.

Multiple neuroprotective mechanisms have been proposed to underlie the beneficial effects of exercise on reducing A$\upbeta$ burden and improving learning and memory in AD animal models. Within the context of CNS lymphatic clearance, this process integrates the upstream glymphatic system with the downstream mLym pathway. A critical regulator of glymphatic CSF flow is the AQP4 water channel, which is highly enriched at astrocytic endfeet. In the present study, however, exercise did not alter total cortical AQP4 protein levels in 5xFAD mice. Although this result may appear inconsistent with previous reports, growing evidence indicates that glymphatic efficiency depends primarily on the polarization of AQP4 at perivascular endfeet rather than on its overall expression level [15, 32]. Accordingly, AQP4 mislocalization can impair glymphatic clearance even when total protein levels remain unchanged [43], whereas exercise has been shown to restore AQP4 polarization and thereby facilitate A$\upbeta$ removal [15, 32]. Beyond CNS lymphatic clearance, exercise is also known to modulate microglial activation, promoting a shift toward an M2-like phenotype that enhances A$\upbeta$ phagocytosis while attenuating neuroinflammation [44, 45]. In addition, A$\upbeta$ can be cleared via transcytosis across the blood-brain barrier (BBB), a process largely mediated by endothelial lipoprotein receptor-related protein in cooperation with P-glycoprotein [46–48]. Exercise improves vascular and endothelial function and increases lipoprotein receptor-related protein 1 expression at the BBB, further supporting A$\upbeta$ efflux in AD models [14, 49]. Despite the involvement of these complementary pathways, our lymphatic vessel ligation experiments demonstrate that the mLym system is indispensable for mediating the beneficial effects of exercise. These results indicate that, even when glymphatic transport, microglial activation, and BBB-mediated efflux are preserved, an intact mLym pathway is required as the terminal route for waste removal from the CNS. Taken together, although exercise engages multiple A$\upbeta$ clearance mechanisms, our findings provide causal evidence that the mLym system represents a critical and non-redundant pathway through which exercise protects against A$\upbeta$ accumulation and cognitive decline.

The 5xFAD transgenes and associated overproduction of A$\upbeta$ negatively impacts mLym integrity in an age-dependent manner. *In vitro*, A$\upbeta$ oligomers (at non-cytotoxic concentrations) reduced VEGFR3 expression in HDLECs, a key receptor promoting lymphangiogenesis. Given that mLym obstruction increased brain A$\upbeta$ load, these findings suggest a bidirectional relationship between impaired mLym function and A$\upbeta$ accumulation. Exercise disrupted this loop by preserving VEGF-C/VEGFR3 signaling, thereby enhancing lymphatic vessel integrity and drainage efficiency.

During exercise, contracting skeletal muscle fibers and other organs release specific peptides, metabolites and RNA species into the circulation; these factors are collectively termed “exerkines” [50]. Among these, miR-142-5p, enriched in EVs during acute exercise, has been shown to promote lymphatic remodeling [51, 52]. In our study, $\text {EV}_{\text {Ex}}$ upregulated CD9 expression in HDLECs, further supporting their role in counteracting A$\upbeta$-induced dysfunction. CD9 is one of the most abundant proteins found in EVs and plays a key role in promoting VEGFR3 signaling; its inhibition has been shown to impair lymphangiogenesis both *in vitro* and *in vivo* [53–55]. While these data implicate exercise-derived EVs in mLym maintenance, the exact molecular contributors remain to be identified.

Consistent with our findings, previous studies have shown that swimming increases levels of VEGF-C, VEGF-D and VEGFR3 in mouse heart and induces cardiac lymphangiogenesis [56]. Additionally, acute exercise elevates the expression level of VEGF-C but not VEGFR3 in leg muscles [57]. These findings collectively indicate that exercise enhances lymphangiogenesis across tissues, likely contributing to improved A$\upbeta$ clearance.

**Fig. 7:**
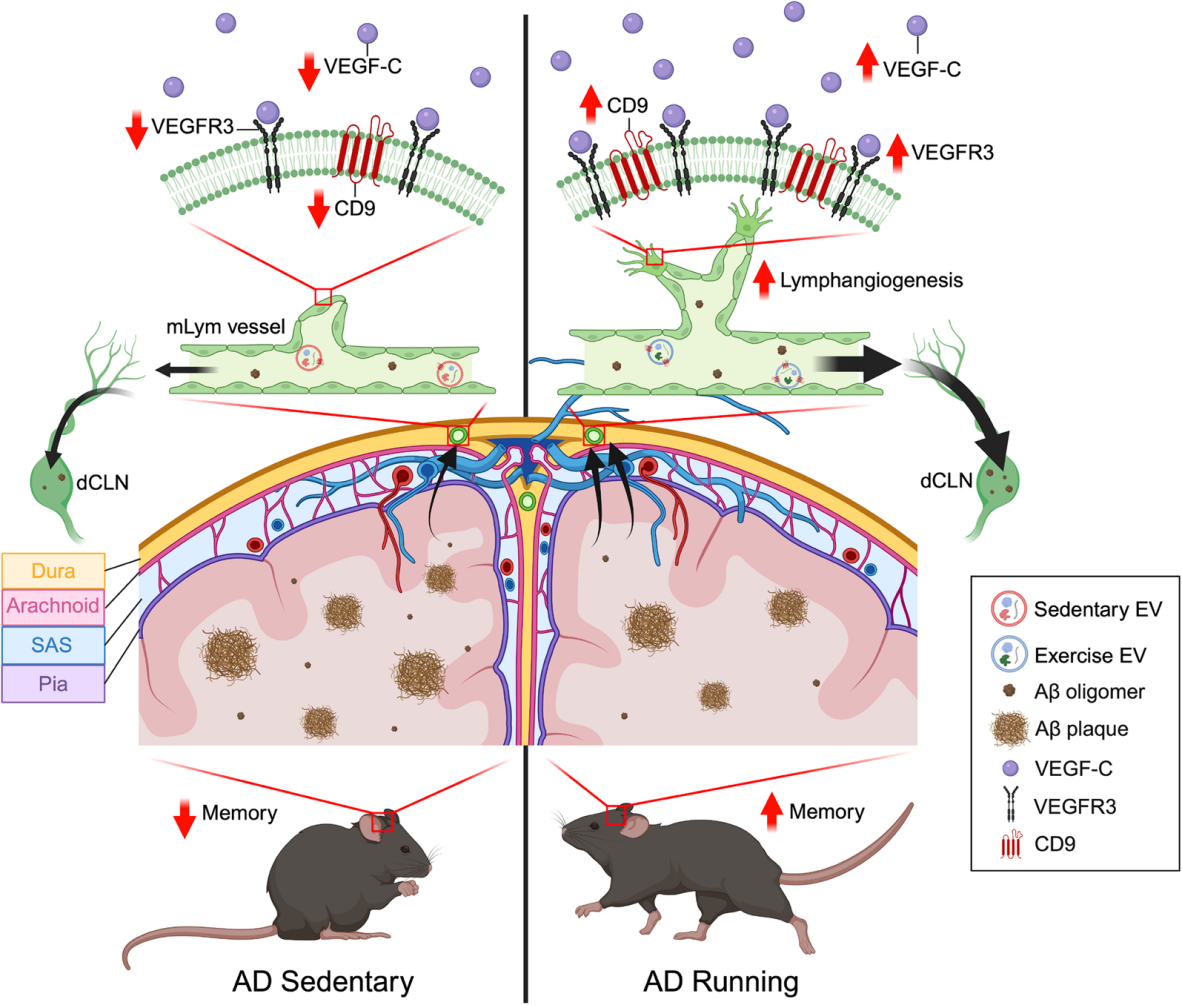
Exercise preserves meningeal lymphatic structure and function, reducing amyloidosis and improving cognition in 5xFAD mice. HFUS imaging with FePt@PLGA nanoparticles enabled real-time visualization of CSF drainage to deep cervical lymph nodes. In sedentary 5xFAD mice, aging was associated with impaired mLym function, reduced VEGF-C/VEGFR3 signaling, increased A$\upbeta$ deposition, and cognitive decline. Surgical ligation of mLym vessels further accelerated amyloid accumulation. In contrast, a 3-month voluntary running regimen restored lymphatic vessel morphology, enhanced CSF clearance, and upregulated VEGF-C and VEGFR3 expression. Exercise also increased levels of CD9-enriched EVs, which counteracted A$\upbeta$-induced suppression of VEGFR3 in lymphatic endothelial cells. These findings support a model in which physical activity enhances mLym-mediated waste clearance and mitigates AD pathology via VEGF-C/VEGFR3 and exerkine-based mechanisms. SAS: subarachnoid space

### Limitation

This study demonstrates the protective effects of running exercise on the mLym system and AD-related pathologies. However, several limitations should be acknowledged. First, we did not assess the effects of exercise in WT mice, which precludes a direct distinction between general exercise effects and genotype-specific responses. Previous studies have consistently shown that exercise in WT mice enhances hippocampal neurogenesis and synaptic plasticity and improves spatial learning and memory [58–60]. In addition, exercise has been reported to augment CSF-interstitial solute transport through both glymphatic and mLym pathways in humans and animal models [11, 32, 33, 61], indicating that the beneficial effects of exercise in WT conditions are well established. Accordingly, our study focused on 5xFAD mice to specifically interrogate AD-related mechanisms. Future studies incorporating exercised WT controls will be important to further delineate genotype-independent and disease-specific effects of exercise. Next, our results lack of the protein expression of LYVE-1, VEGF-C, and VEGFR3 to complement the mRNA expression data. According to the limited available sample volume and mLym tissue, they were not sufficient to conduct protein assays in parallel with mRNA analysis. Hence, we prioritized mRNA expression, which allowed us to reliably assess changes in gene regulation related to lymphatic function. Nonetheless, these parts of data should be fully investigated in future studies.

## Conclusion

This study provides compelling evidence that voluntary exercise strengthens the brain’s lymphatic clearance system in an AD mouse model. Exercise preserves the structure and function of mLym vessels, leading to reduced A$\upbeta$ accumulation and improved cognitive performance. The lymphatic vessel blocking abolishes these exercise benefits. At the molecular level, exercise mitigates A$\upbeta$-induced suppression of VEGF-C/VEGFR3 signaling and delivers protective exerkines, such as CD9, via EVs (Fig. 7). These findings highlight the potential of enhancing lymphatic drainage, either through regular physical activity or VEGF-C-based interventions, as a therapeutic approach to delay or prevent AD progression. Future research should focus on identifying the specific EV cargo involved and explore the translational potential of these findings in human subjects.

## Supplementary Information

Supplementary Material 1

## Data Availability

Not applicable

## Abbreviations

AD: Alzheimer’s disease
A$$\upbeta $$: Amyloid plaque
ANOVA: Analysis of variance
AQP4: Aquaporin-4
CSF: Cerebrospinal fluid
dCLNs: Deep cervical lymph nodes
EGM-MV2: Endothelial growth medium
Ex: Running exercise
EVs: Extracellular vesicles
FePt@PLGA: Iron-platinum nanoparticles in poly(lactic-co-glycolic acid) nanocarriers
HDLECs: Human dermal lymphatic endothelial cells
HFUS: High frequency ultrasound
ICV: Intracerebroventricular
LYVE-1: Lymphatic vessel endothelial hyaluronan receptor 1
mLym: Meningeal lymphatic
PBS: Phosphate buffered saline
qPCR: Quantitative polymerase chain reaction
Sed: Sedentary
SSS: Superior sagittal sinus
TS: Transverse sinus
VEGF-C: Vascular endothelial growth factor C
VEGFR3: Vascular endothelial growth factor receptor 3
WT: Wild type
