# Supplementary material for: Running exercise mitigates amyloidosis in 5xFAD mice by improving the structure and function of the meningeal lymphatic system
Source: Acta Neuropathol Commun. 2026 Jan 15;14:126. doi: 10.1186/s40478-025-02218-2 (PMC13255411; doi:10.1186/s40478-025-02218-2)
Supplement: Supplementary file 1 — Supplementary Material 1 [file 40478_2025_2218_MOESM1_ESM.pdf]

Supplementary figures and legends

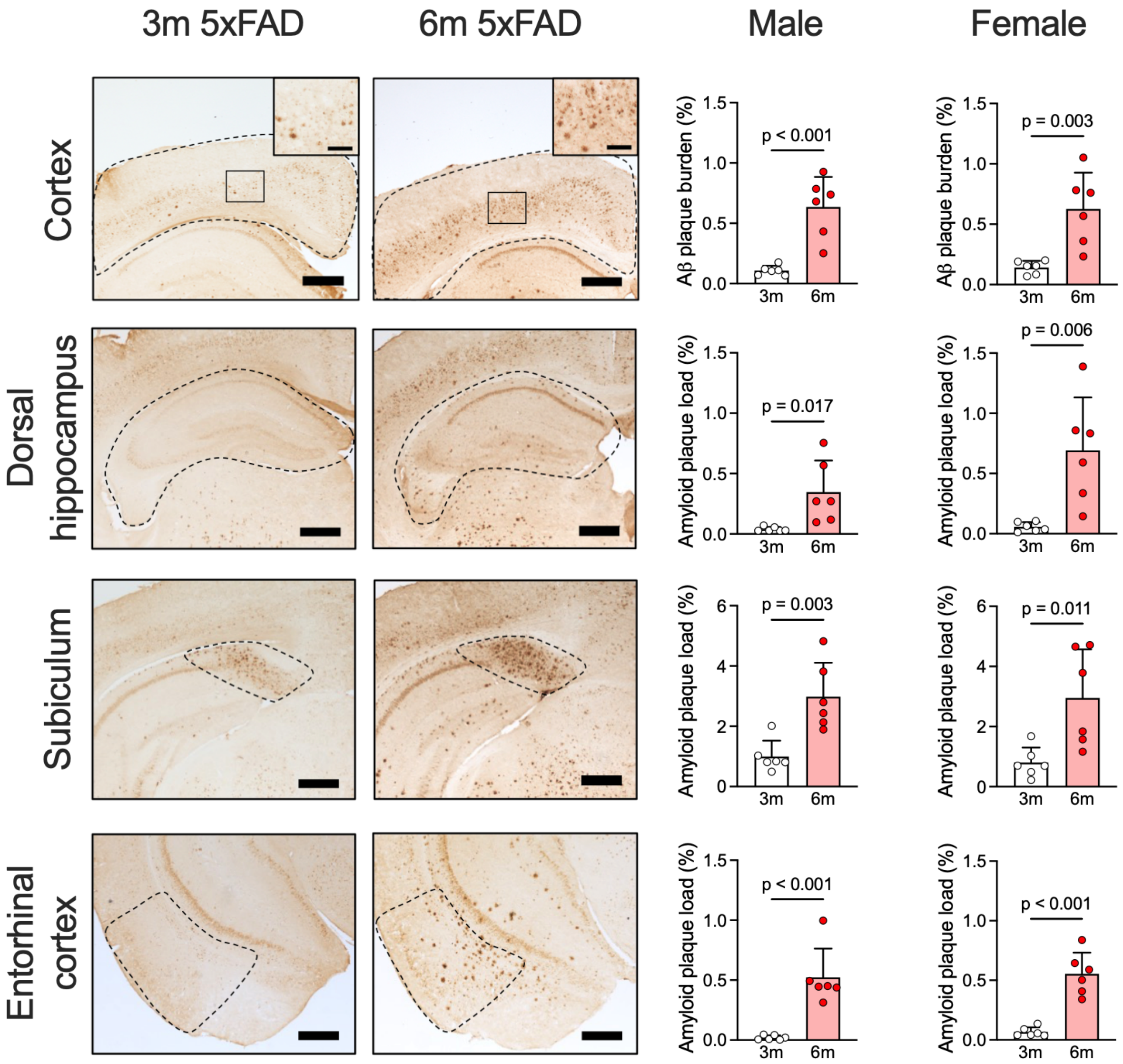

Figure S1. Age- and sex-dependent amyloid plaque deposition in 5xFAD mouse brains.
Left Panels: Representative micrographs of Aβ immunostaining in the cortex, dorsal hippocampus, subiculum, and entorhinal cortex of 3- and 6-month-old 5xFAD mice. Scale bar = 500 µm. Right Panels: Quantitative analysis of Aβ plaque burden in male (n = 6) and female (n = 6) mice at each age. Unpaired two-tailed Student t-test.

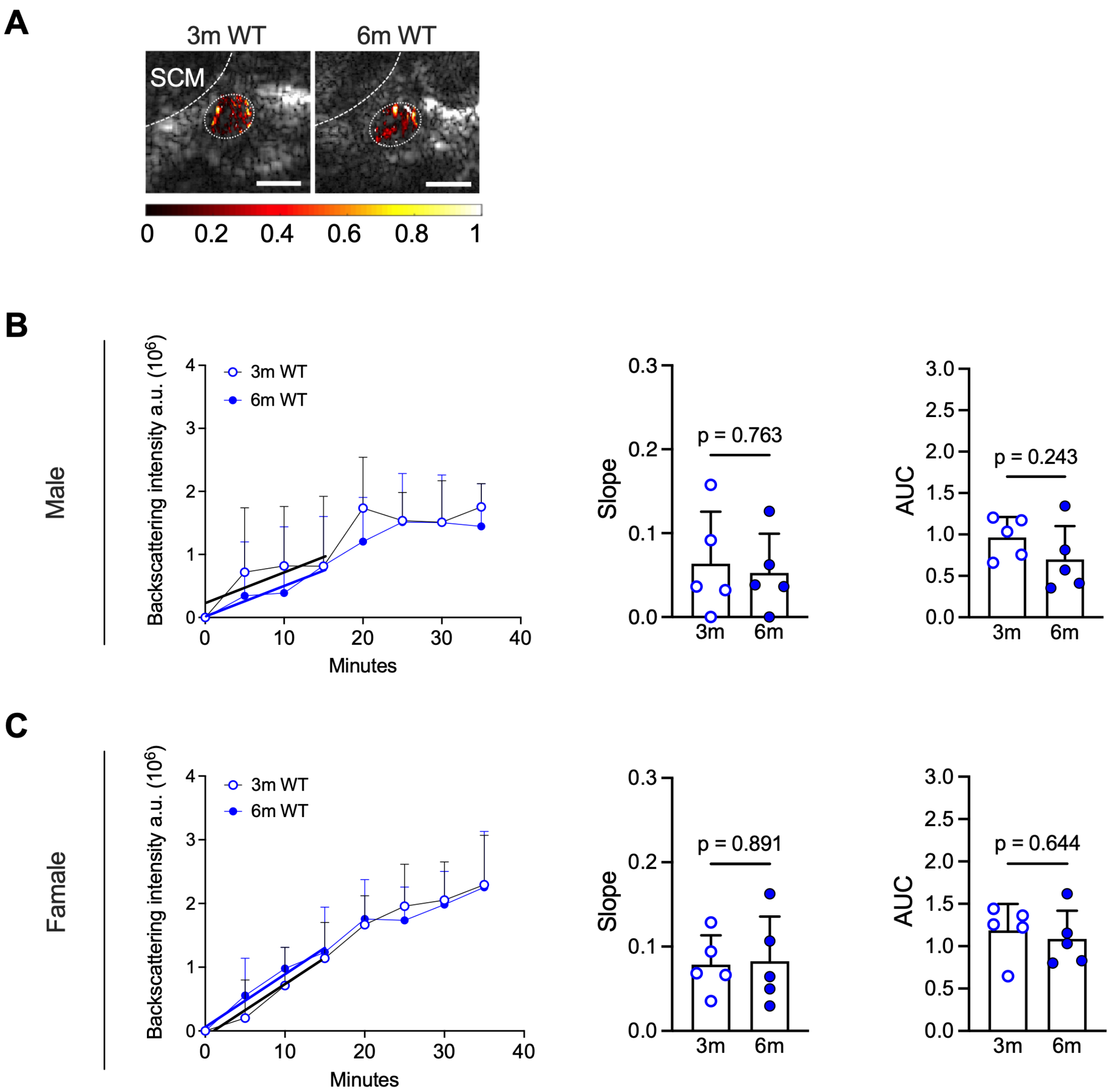

Figure S2. Age- and sex-dependent analysis of meningeal lymphatic function in C57BL/6J wild-type mice.

A Representative HFUS Imaging. High-frequency ultrasound (HFUS) images displaying nanoparticle signal mapping within the deep cervical lymph node (dCLN) region (indicated by dashed circles) in 3- and 6-month-old wild-type (WT) C57BL/6J mice, captured 35 minutes post-intracerebroventricular injection. SCM = sternocleidomastoid muscle. Scale bar = 1 mm. The accompanying color bar denotes the normalized intensity of backscattered signals. **B**, C HFUS Signal Analysis. Left panels present averaged HFUS signal intensity traces from 0 to 35 minutes and corresponding slopes from 0 to 15 minutes (straight lines) in male **(B)** and female **(C)** WT mice. Right panels display quantitative analyses of the slope and area under the curve (AUC) metrics. N = 5. Unpaired two-tailed Student's t-test.

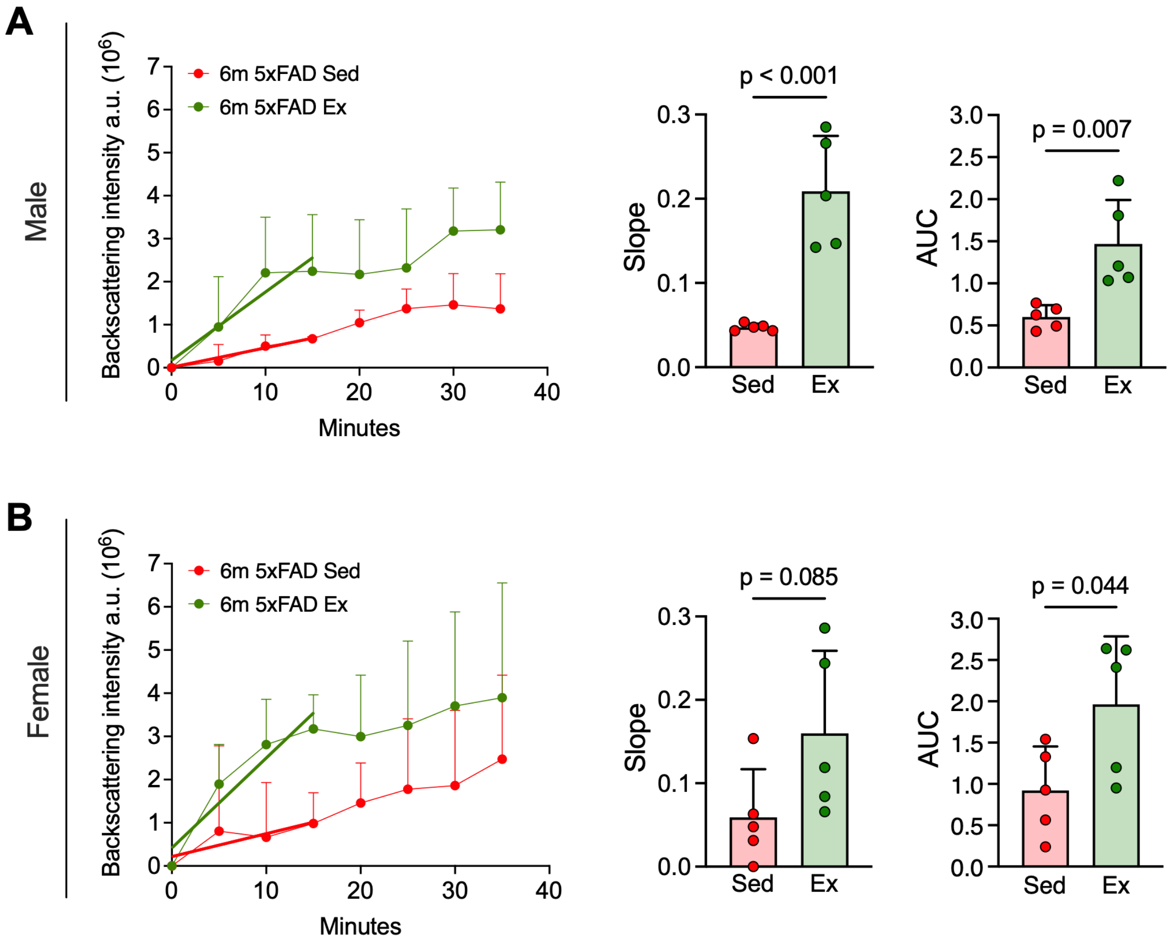

Figure S3. Exercise enhances meningeal lymphatic function in 6-month-old male and female 5xFAD mice.

A, B Averaged high-frequency ultrasound signal intensity traces (0-35 min) and corresponding slopes (0-15 min) in 6-month-old male **(A)** and female **(B)** 5xFAD mice, with or without a 3-month Ex intervention. Quantitative results are shown on their respective right panels. N = 5. Unpaired two-tailed Student t-test.

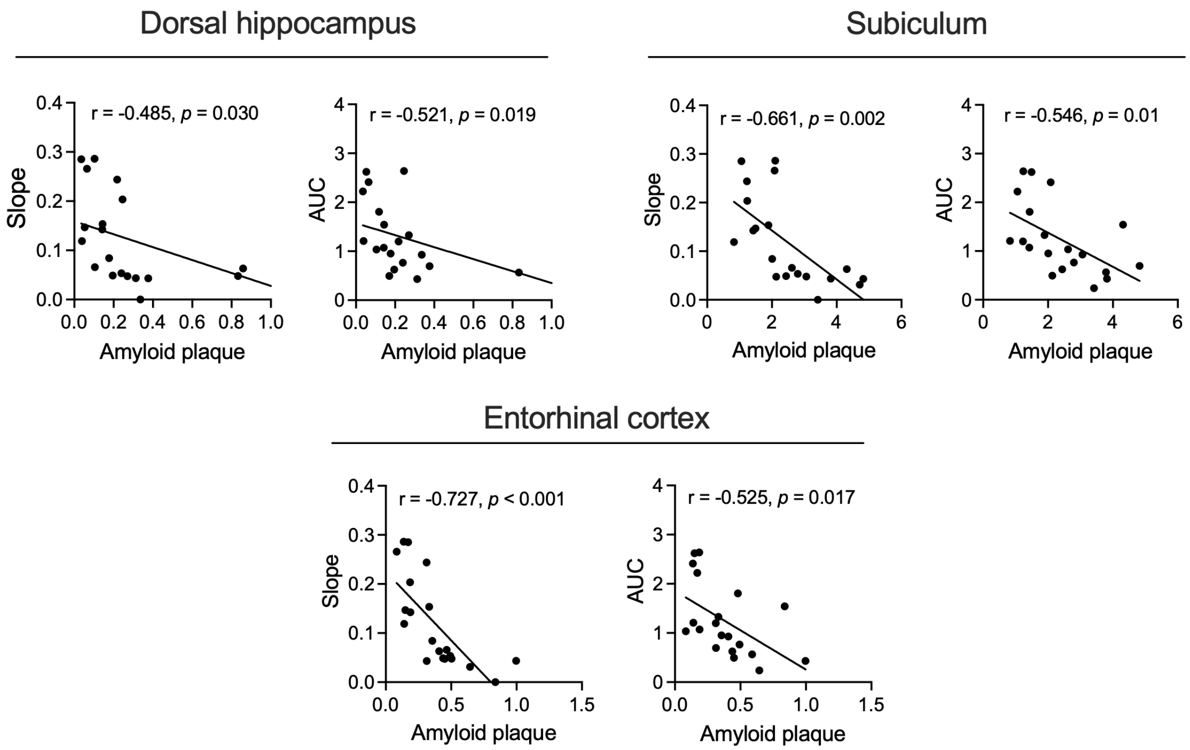

Figure S4. Association between meningeal lymphatic function and amyloid plaque load in 6-month-old 5xFAD mice.
Correlation analyses between amyloid plaque load and either the slope (0-15 min) or the area under the curve (AUC, 0-35 min) of HFUS signal traces in the dorsal hippocampus, subiculum, and entorhinal cortex of 6-month-old 5xFAD mice.

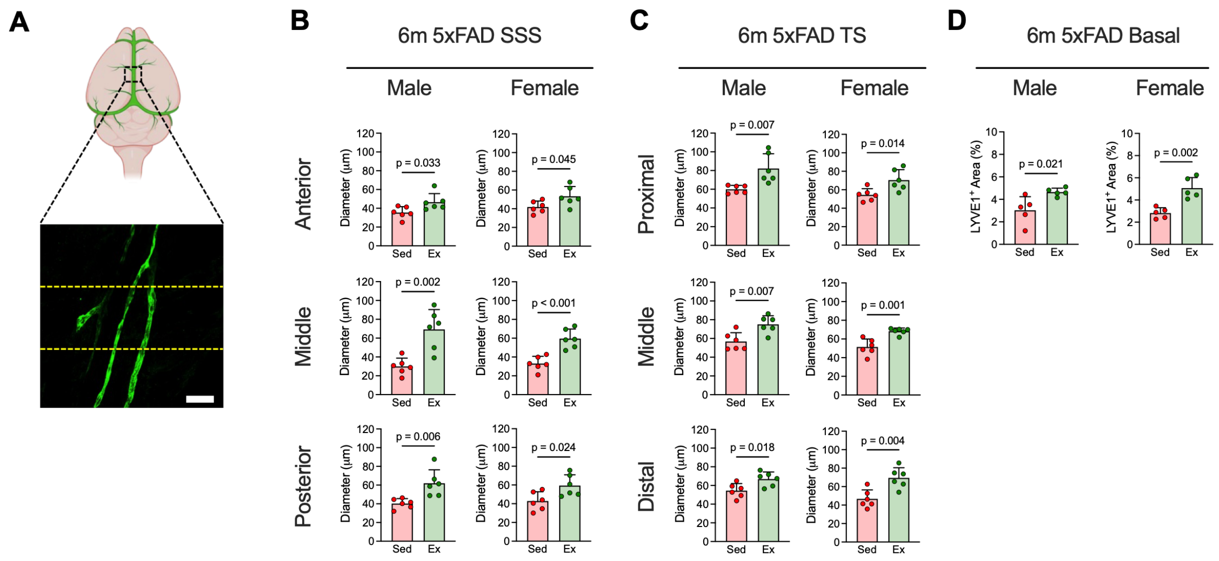

Figure S5. Exercise enhances lymphangiogenesis at the superior sagittal sinus (SSS) and transverse sinus (TS) in 6-month-old male and female 5xFAD mice. **A** Schematic representation of meningeal lymphatic (mLym) vessel diameter measurements. The enlarged image shows diameter quantification points, marked by perpendicular lines to the vessel flow direction at 150 μm intervals (yellow dashed lines). **B** Quantification of mLym vessel diameters along the anterior, middle, and posterior regions of the SSS in 6-month-old sedentary (Sed) and exercised (Ex) male and female 5xFAD mice ($n = 6$ per group). **C** Quantitative results of mLym vessel diameters along the proximal, middle, and distal regions of the TS in the same groups. **D** Quantitative results of LYVE-1^+ signal area (%) in the basal meninges of 6-month-old 5xFAD Sed and Ex mice. Unpaired two-tailed Student t-test. Combined male and female data are shown in Fig. 3.

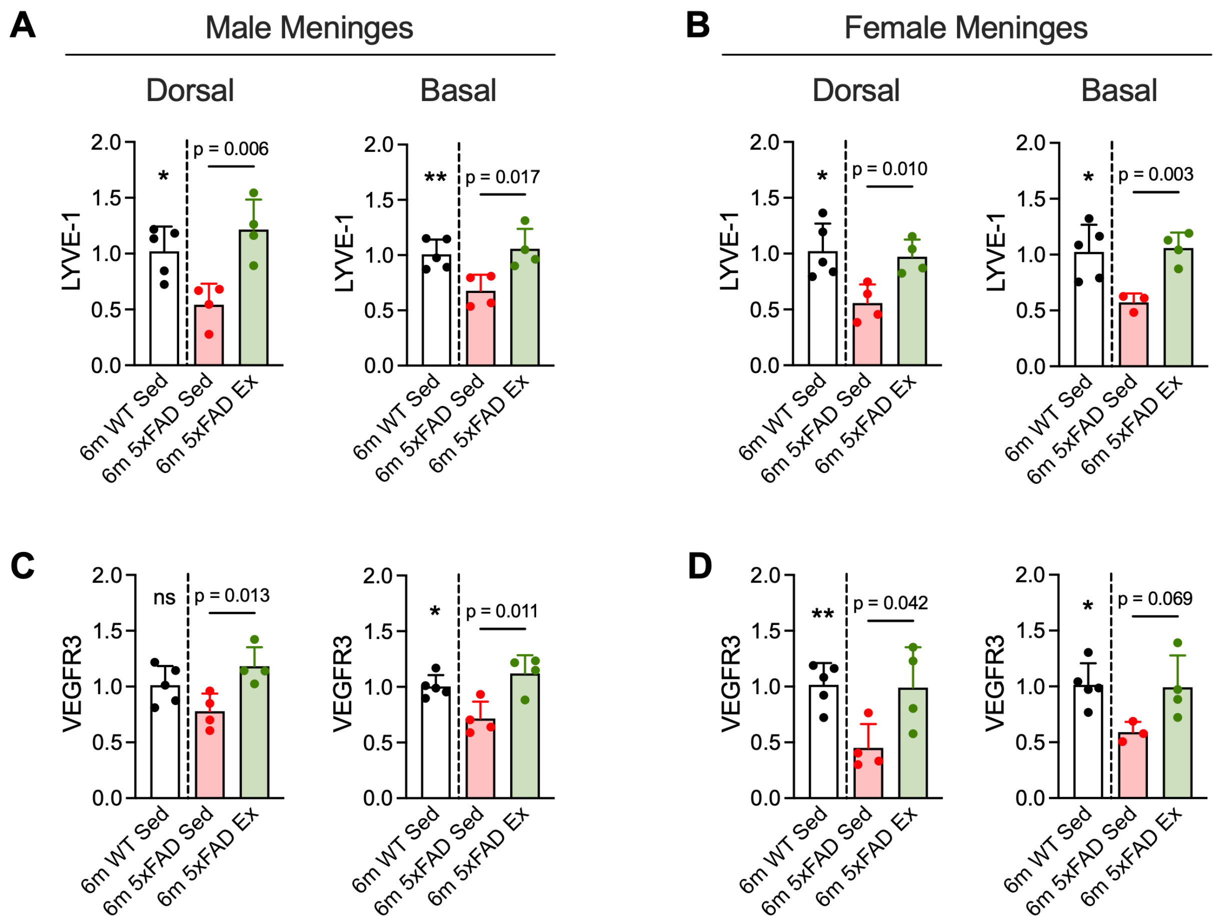

Figure S6. The lymphatic vessel-related gene expression in meninges of 6-month-old male and female 5xFAD mice with or without Ex.

A, B Quantitative results of LYVE-1 expression in the dorsal and basal parts of meninges in male **(A)** and female **(B)** 5xFAD mice with or without Ex. **C, D** Quantitative results of VEGFR3 expression in the dorsal and basal parts of meninges in male **(C)** and female **(D)** 5xFAD mice with or without Ex. For all quantitative results, the aged-matched wild-type (WT) littermate group was shown as a reference. * $p < 0.05$, ** $p < 0.01$ compared with 5xFAD Sed group (WT vs. Sed). ns = not significant. Unpaired two-tailed Student t-test.

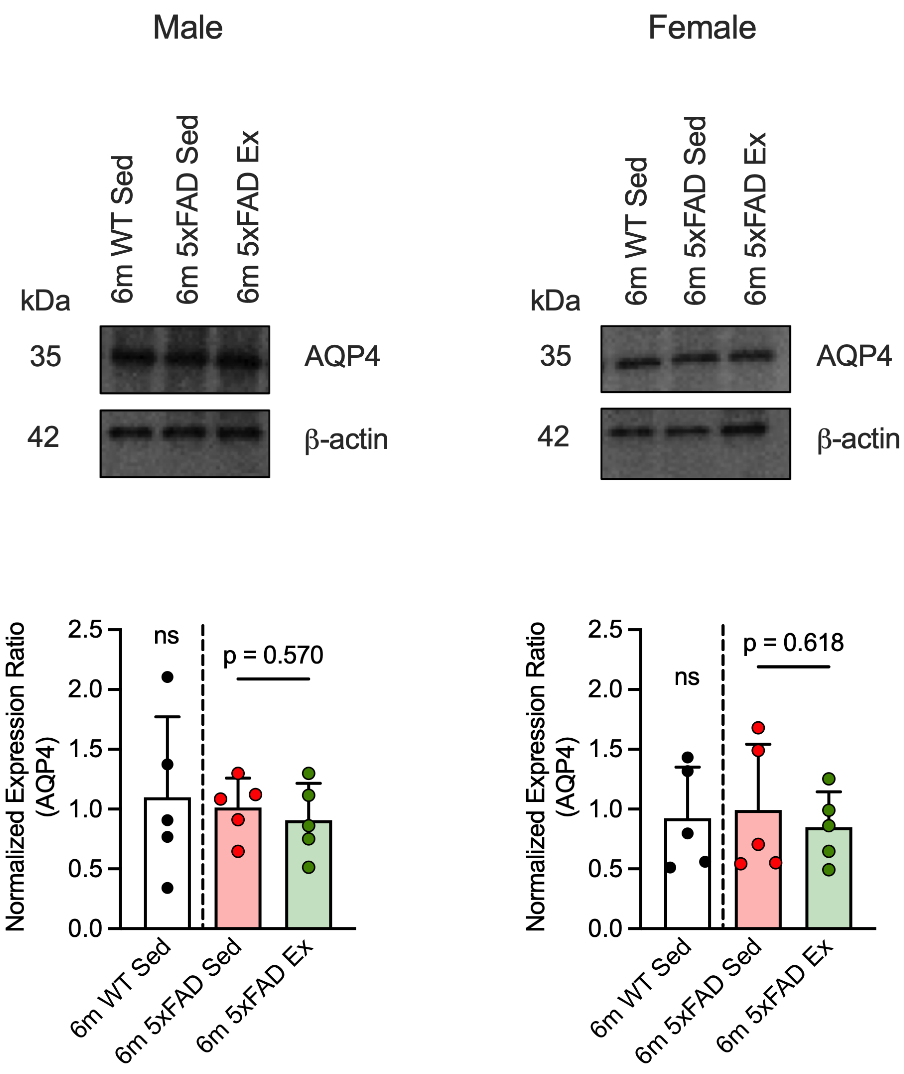

Figure S7. Cortical AQP4 expression in 6-month-old male and female 5xFAD mice with or without Ex.
Immunoblot and quantitative results of AQP4 protein expression in the cortex of male and female WT and 5xFAD mice. N = 5. Unpaired two-tailed Student's t-test. The age-matched wild-type (WT) littermate group was used as a reference to demonstrate the effect of the 5xFAD transgene (WT Sed vs. 5xFAD Sed; ns = not significant).

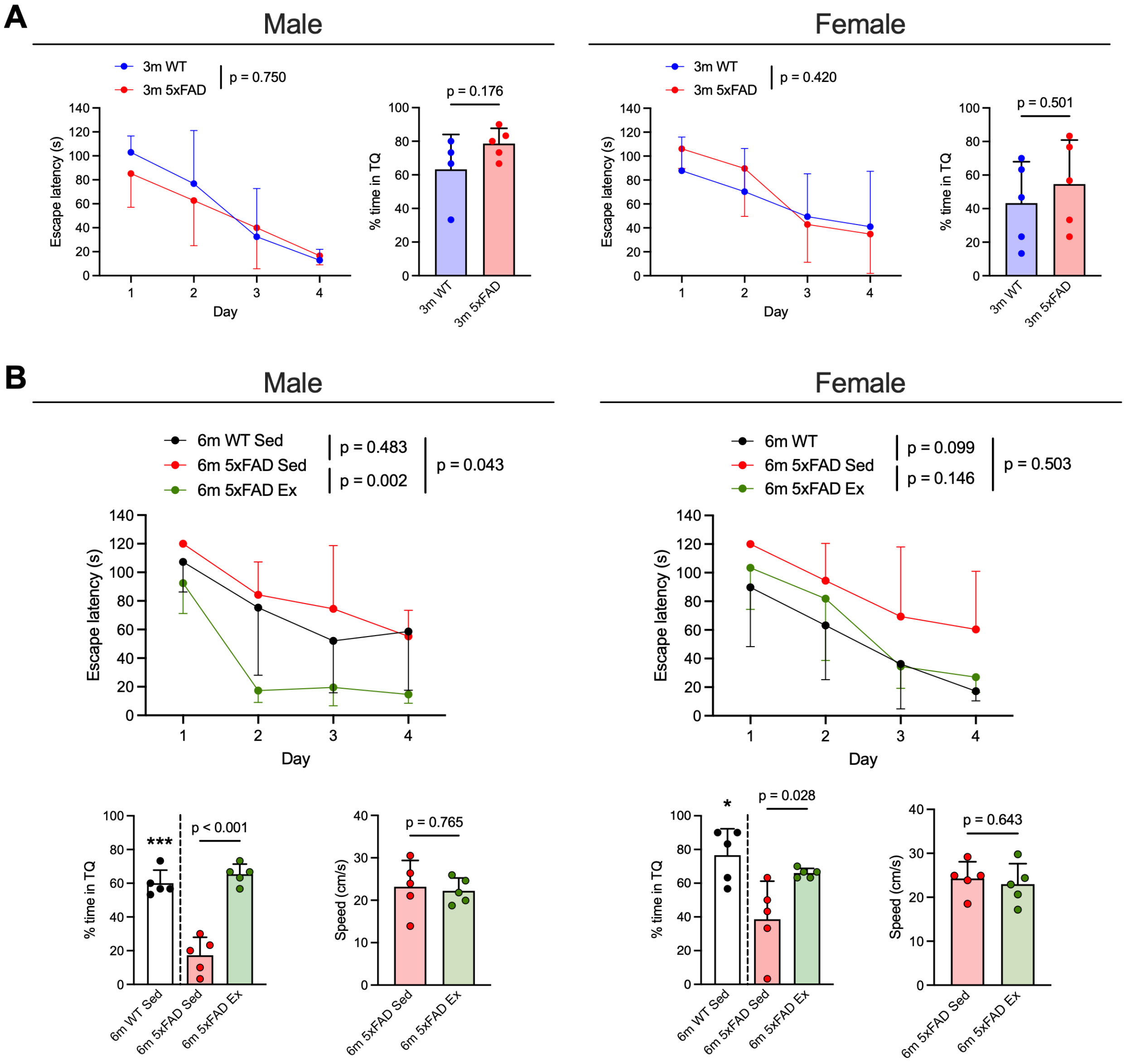

Figure S8. Learning and memory performances of 3- and 6-month-old male and female 5xFAD mice with or without Ex.

A Trends of escape latency of 3-month-old male and female WT and 5xFAD mice. N = 5. Repeated measures two-way ANOVA. Quantitative results of percent time in target quadrant are shown on the right side of graphs. N = 5. Unpaired two-tailed Student t-test. **B** Trends of escape latency of 6-month-old male and female WT, 5xFAD Sed, and 5xFAD Ex mice. N = 5. Repeated measures two-way ANOVA. Quantitative results of percent time in target quadrant (left) and swim speed (right) are shown below the graphs. N = 5. Unpaired two-tailed Student t-test. The aged-matched wild-type (WT) littermate group was shown as a reference. * $p < 0.05$, *** $p < 0.001$ compared with 5xFAD Sed group (WT vs. Sed), Unpaired two-tailed Student t-test.

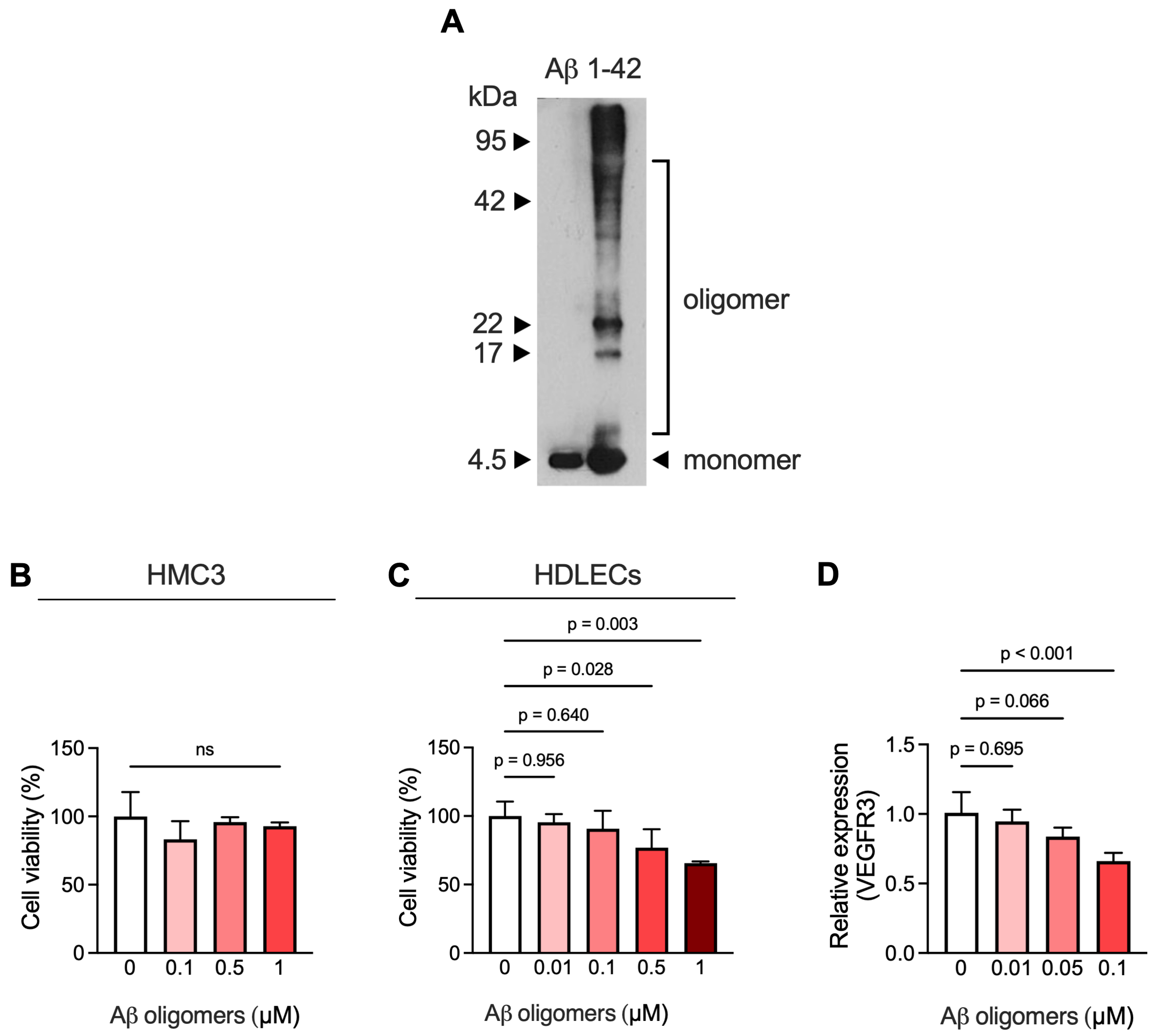

Figure S9. Concentration-dependent effect of Aβ oligomer treatment on HMC3 and HDLECs cell viability.
A Immunoblot analysis confirming the presence of Aβ oligomers. **B** Quantification of HMC3 microglial cell viability following treatment with increasing concentrations of Aβ oligomers. ns = not significant. **C** Quantification of HDLEC (human dermal lymphatic endothelial cell) viability under the same treatment conditions. **D** Quantitative results of VEGFR3 gene expression in response to gradient concentration of Aβ oligomers. One-way ANOVA.

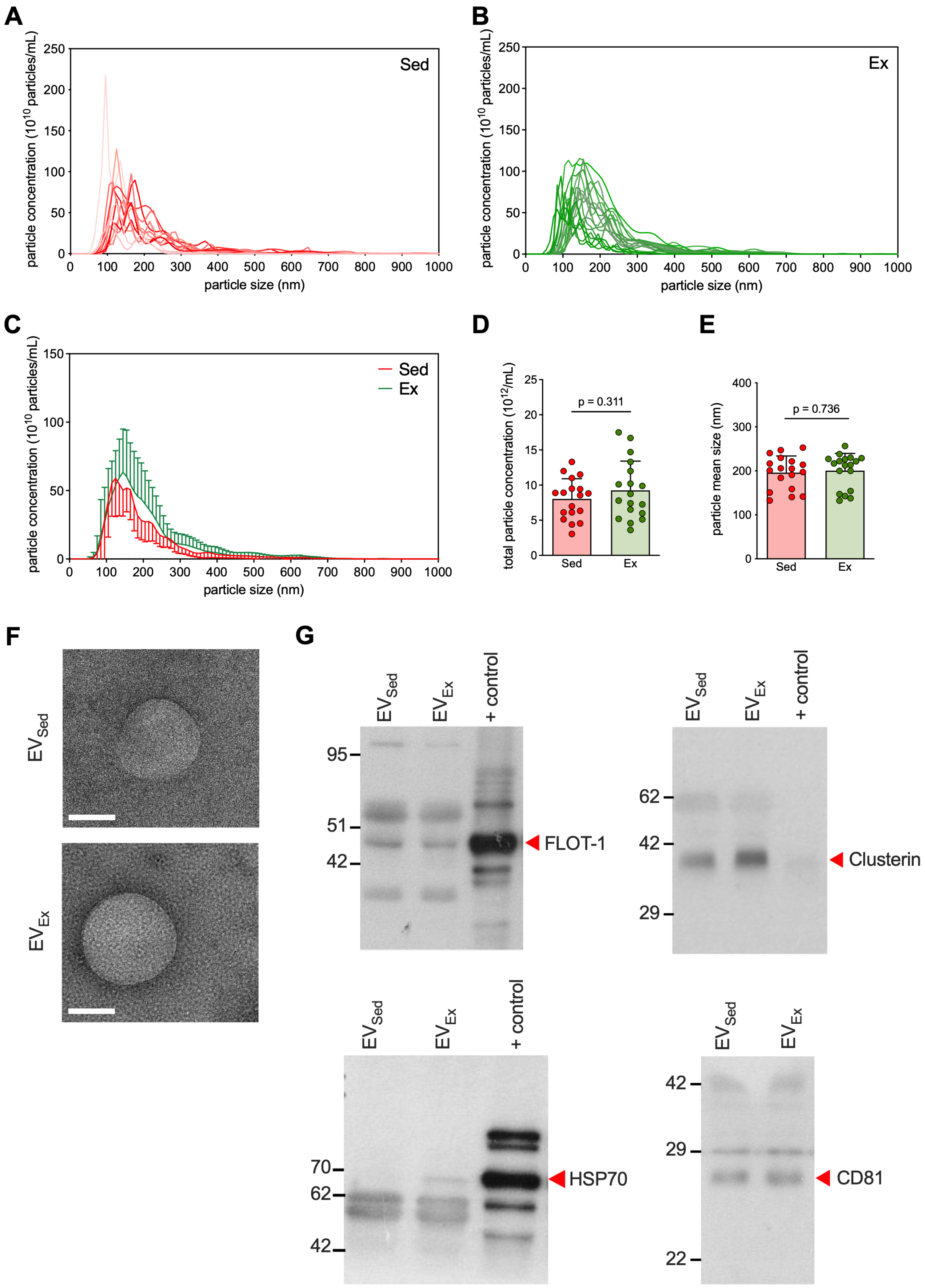

Figure S10. Comprehensive analysis of plasma-derived extracellular vesicles (EVs) from sedentary and exercised rats.

A, B Nanoparticle tracking analysis profiles displaying particle concentrations in EV samples from Sed **(A)** and Ex **(B)** rats. Each trace corresponds to an individual sample from a

single rat. **C, D** Quantitative assessment of total particle concentrations in EVs from Sed and Ex rats. **E** Quantitative evaluation of the mean particle size in EVs from both groups. **F** Representative cryo-transmission electron microscopy images illustrating the morphology of circulating EVs from Sed and Ex rats. Scale bar = 100 nm. **G** Representative Western blot images detecting EV markers: FLOT-1, HSP70, Clusterin, and CD81. Data in panel **C** are presented as mean ± standard deviation and analyzed using a mixed-model two-way ANOVA followed by Tukey's multiple comparisons test. Data in panels **D** and **E** are expressed as median with interquartile range and analyzed using the Mann-Whitney U test. n = 18 for panels **A**-**E**.
